# Polysaccharides as Protective Agents against Heavy Metal Toxicity

**DOI:** 10.3390/foods13060853

**Published:** 2024-03-11

**Authors:** Lukman Iddrisu, Felix Danso, Kit-Leong Cheong, Zhijia Fang, Saiyi Zhong

**Affiliations:** 1 College of Food Science and Technology, Guangdong Provincial Key Laboratory of Aquatic Product Processing and Safety, Guangdong Provincial Engineering Technology, Research Center of Marine Food, Key Laboratory of Advanced Processing of Aquatic Products of Guangdong Higher Education Institution, Guangdong Ocean University, Zhanjiang 524088, China; lukmaniddrisu54@gmail.com (L.I.); klcheong@gdou.edu.cn (K.-L.C.); zhongsy@gdou.edu.cn (S.Z.); 2 Department of Veterinary Medicine, Guangdong Ocean University, Zhanjiang 524088, China; fdanfreeman@gmail.com

**Keywords:** polysaccharides, heavy metals, alleviation, chelating agent, microbiota

## Abstract

Polysaccharides are functional foods or drugs that can be used to alleviate heavy metal poisoning by cadmium, lead, mercury, and arsenic. Industries generate substantial quantities of toxic heavy metal wastes, such as wastewater discharges, paints, electronic waste, batteries, pigments, and plastics, into the environment that pose a risk to human health. Therefore, it is imperative to eliminate accumulated heavy metal ions from the body and the environment. Heavy metal toxicity can lead to decreased energy levels and impair the functioning of vital organs, such as the brain, lungs, kidneys, liver, and blood. Prolonged exposure can result in progressive physical, muscular, and neurological degeneration that resembles conditions such as multiple sclerosis, Parkinson’s disease, Alzheimer’s disease, and muscular dystrophy. Polysaccharides operate through mechanisms such as chelation, antioxidant defense, immunomodulation, and tissue repair. Polysaccharides involved in heavy metal removal include methionine and cysteine, together with N-acetylcysteine, an acetylated form of cysteine, S-adenosylmethionine, a metabolite of methionine, α-lipoic acid, and the tripeptide glutathione (GSH). These compounds effectively bind with harmful heavy metals to create a stable complex and defend biological targets from metal ions, thus decreasing their harmful effects and causing them to be excreted from the body. This review also highlights the importance of polysaccharides’ ability to mitigate oxidative stress, enhance immune responses, and support tissue repair processes. Polysaccharides are ubiquitous in nature and take part in diverse processes, making them potential natural therapies for heavy metal-related diseases. This review discusses the effectiveness of natural polysaccharides and the mechanisms that allow them to bind with heavy metals to alleviate their effects from the body and the environment. Polysaccharides have inherent features that enable them to function as pharmacological agents and regulate the immune response.

## 1. Introduction

Heavy metals are distinct metallic elements and compounds that possess the capacity to pose a threat to human health. Cadmium (Cd), lead (Pb), mercury (Hg), and arsenic (As) are substances that commonly exist in small quantities in the environment. When consumed in higher dosage, they pose a risk to human health. Typically, millions of individuals worldwide are exposed to these metals by ingestion (consumption through eating or drinking) or inhalation (breathing in). Engaging in agricultural activities involving the application of mineral fertilizers, and residing or working close to an industrial site that uses these metals and their compounds increase the possibility of exposure. Similarly, residing near a location where these metals have been inadequately disposed of also poses a risk of exposure [1]. Subsistence, and hunting and gathering activities can also pose a great risk of exposure. Metals may have been the earliest poisons recognized by humans, with lead extraction dating back to 2000 B.C., and arsenic and mercury mentioned by Theophrastus of Eresos and Pliny the Elder [2] whereas cadmium was discovered in 1817 [2] Fewer than 30 metals have been documented to have dangerous effects on humans, with approximately 80 of the 118 elements classified as metals [2]. Heavy metals harm the body by binding to reactive groups necessary for physiological functioning, interacting with oxygen, sulfur, and nitrogen ligands, and forming complexes with proteins, which are influenced by age, development stage, lifestyle, and immunological conditions [3,4,5]. According to epidemiological studies, heavy metal exposure has been linked to chronic diseases like neurological problems, diabetes, cancer, and respiratory, cardiovascular, kidney, and skin ailments [6,7,8].

The principle of chelation is rooted in basic coordination chemistry. However, the development of an optimal chelator and chelation therapy that effectively eliminates a particular harmful metal from a specific location in the body requires a comprehensive approach for drug design. Chelating agents are substances, either organic or inorganic, that have the capacity to bind metal ions and create complex structures known as ‘chelates’. Bidentate chelates have atoms that bind to ligands through either two covalent connections or one covalent and one coordinate linkage. Chelation therapy has traditionally been employed to decrease the accumulation of harmful metals in the bodies of severely symptomatic patients with high biological indicators [9,10,11]. Chelating substances can influence the toxicity of metals by facilitating the movement of the harmful metal into urine. A chelating agent, which can create a stable complex with a harmful metal, can protect biological targets from metal ions, thus decreasing its harmful effects in the surrounding area [12]. The precipitation of metal sulfides is a highly efficient technique for eliminating heavy metal ions. An important benefit of utilizing sulfides is the reduced solubility of metal compounds in comparison to hydroxide precipitates [13,14,15,16].

The primary way to address heavy metal poisoning is to bind and alleviate accumulated heavy metals from the human body and reduce their toxic impact on organisms. The conventional approach primarily uses polysaccharides, chelating agents, antioxidant defense, and immunomodulation to alleviate heavy metal ions. Polysaccharides possess distinctive chemical bonds and functional groups that enable their effective elimination of heavy metals. Multiple investigations have additionally verified that polysaccharides have the capacity to alleviate heavy metal toxicity [17]. Current research attributes the adsorption mechanism of polysaccharides for heavy metals to the mix of chemical groups (such as hydroxyl, carbonyl, sulfhydryl, etc.) and the unique spatial structure of polysaccharides. This combination allows the polysaccharides to form complexes with other metal ions. Conversely, plant polysaccharides have gained significant attention in studies because of their various biological properties, including antioxidant, hypoglycemic, anti-inflammatory, and gut flora regulation capabilities [18,19,20]; however, there is a scarcity of studies documenting the capacity of polysaccharides derived from plants to adsorb heavy metals, and the underlying mechanism of this adsorption process remains unclear. The potential of polysaccharides to enhance the elimination of toxins from the human body is significant. Several studies have investigated the elimination of heavy metals by using polysaccharides. Nevertheless, when discussing the topic of polysaccharide adsorption of heavy metals, many studies predominantly emphasize the adsorption of heavy metals by fungal polysaccharides [21,22,23,24], bacterial extracellular polysaccharides [25,26], chitosan [27], and others. Immunity, as the body’s inherent defense mechanism, plays a crucial role in combating infectious diseases and regulating inflammation caused by heavy metal toxicity. Individuals with compromised immune systems are susceptible to a range of infections and tumors owing to weakened immune surveillance caused by low immunological function. Specific monosaccharides, such as galactose, mannose, rhamnogalacturonan-I, arabinogalactan, and uronic acid, are strongly linked with immunological enhancement. Chemical alterations of polysaccharides can enhance their biological activity and potentially generate novel functionalities [28].

This review explores the generalizability of the therapeutic effectiveness of polysaccharides in mitigating heavy metal toxicity and promoting recovery from illnesses. It focuses on their roles in chelation, antioxidant defense, immunomodulation, and tissue repair, emphasizing the need for further research. Polysaccharides have inherent features that enable them to function as pharmacological agents and regulate immune responses.

## 2. Characteristics of Polysaccharides

**Figure 1:**
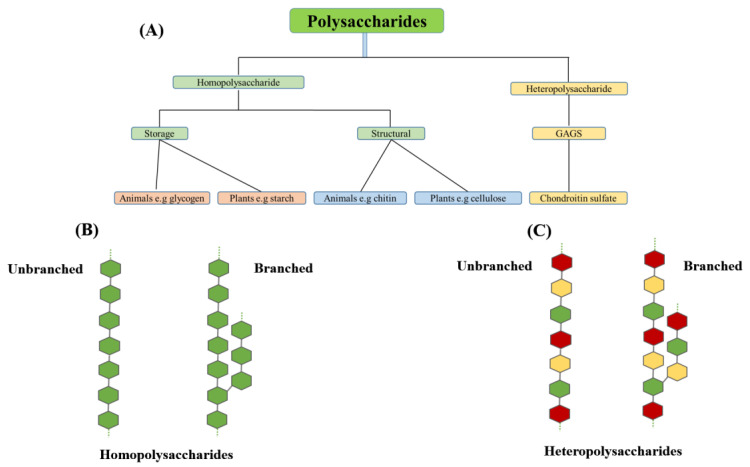
Classification of polysaccharides (**A**), homopolysaccharides (**B**), and heteropolysaccharides (**C**) of different types of natural polysaccharides. Note: GAGS means Glycosaminoglycan.

Polysaccharides are macromolecules that undergo a wide range of structural modifications, making them extremely interesting and adaptable in terms of their functions. Their composition has a far higher level of complexity than other naturally occurring macromolecules, such as proteins and nucleic acids. Polysaccharides are composed of more than ten monomers, referred to as monosaccharides, which can vary in number from eleven to several thousand. Polysaccharides can be composed of either one type of monomer (homoglycans) or numerous types of monomers (heteroglycans), and each unit can be connected to the others in various ways. As a result, they can arrange themselves in either linear or branched structures, form circular shapes, and include components such as proteins and lipids. Thus, it can be inferred that an unlimited array of polysaccharide structures exists (Figure 1) [29,30].

Polysaccharides are foods or drugs that offer a broad array of applications and advantages in diverse sectors. Their economic advantages include the following: (1) Polysaccharides are common in nature and can be obtained from renewable plant and microbial sources, decreasing dependence on non-renewable resources. (2) Polysaccharides are more cost-effective than their synthetic counterparts, which enhances their appeal for industrial use. Their pharmaceutical and medical advantages include the following: (1) Polysaccharides can serve as medication transporters, safeguard medicines from degradation, and facilitate regulated release. (2) Polysaccharides have wound-healing properties and can be utilized in dressings and ointments for wound treatment. Their food benefits include the following: (1) Polysaccharides can serve as fat substitutes in low-fat food items, enhancing their nutritional composition. (2) Specific polysaccharides function as prebiotics, stimulating the proliferation of beneficial gut bacteria and enhancing gut health. (3) Polysaccharides are utilized in the food industries to enhance the texture, stability, and shelf life of food items.

*Homopolysaccharides*

- Contain a single type of monomer.
- Storage of monomer: glycogen in animals; starch in plants.
- Structural elements: cellulose in plants, chitin.

*Heteropolysaccharides*

- Contain two or more types of monomer.
- Extracellular support.
- Bacterial cell wall.
- Extracellular matrix of animals.

Natural polysaccharides are frequently used to develop solid pharmaceutical formulations. They are affordable and easily accessible from a diversity of sources including plants, animals, microbes, and marine species. Polysaccharides exhibit a diverse array of qualities and possess excellent stability, safety, low toxicity, and a broad variety of solubility characteristics [31]. Polysaccharides in organisms serve as both a source of energy for cellular processes and structural elements that are often hydrophilic and can be broken down by enzymes. Polysaccharides display a remarkable range of characteristics as a result of distinct variations in their structural arrangement, including the composition of monosaccharide units, the connections between chains, the length of the chains, and their shapes. These factors affect properties such as solubility, the ability to form gels, flow behavior, and surface characteristics [32].

Polysaccharides are the predominant type of carbohydrate found in nature. They are characterized by their chemical structure, which consists of units of monosaccharides linked with glycosidic bonds. These units can be either sugar residues linked together or covalently bonded to other structures, such as peptides, amino acids, and lipids.

### 2.1. Sources of Polysaccharides

**Figure 2:**
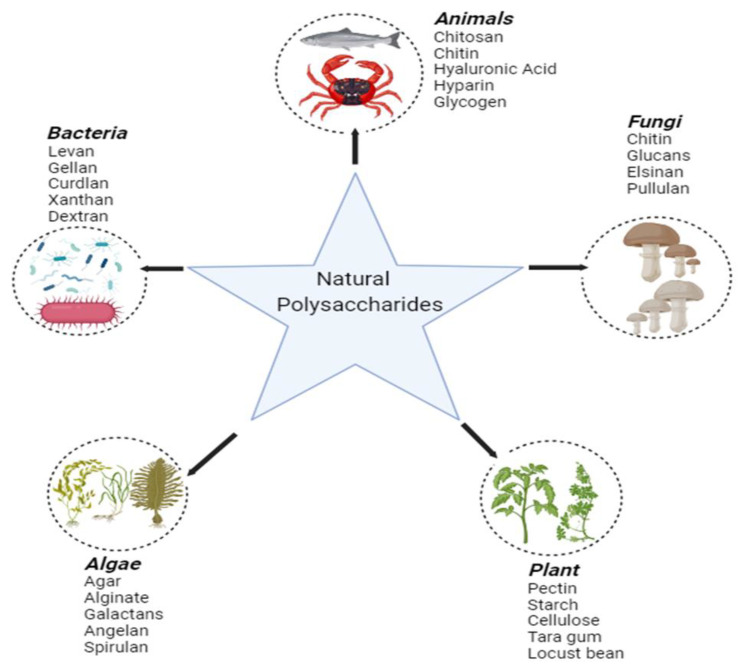
Natural polysaccharide sources.

Polysaccharides of natural origin can be derived from diversified sources (Figure 2), such as algae (e.g., alginate), plants (e.g., pectin, gums), microbes (e.g., dextran), and animals (e.g., chitosan) [33,34], and can be regarded as crucial functional substances that perform significant functions in several physiological and biological processes, such as antioxidant, anticancer, anti-hyperglycemic, and immunological regulatory activities [35,36]. The utilization of microbial polysaccharides in biotechnology and biological sciences began several years ago [35,36].

### 2.2. Structural Diversity of Polysaccharides

#### 2.2.1. Cellulose

**Figure 3:**
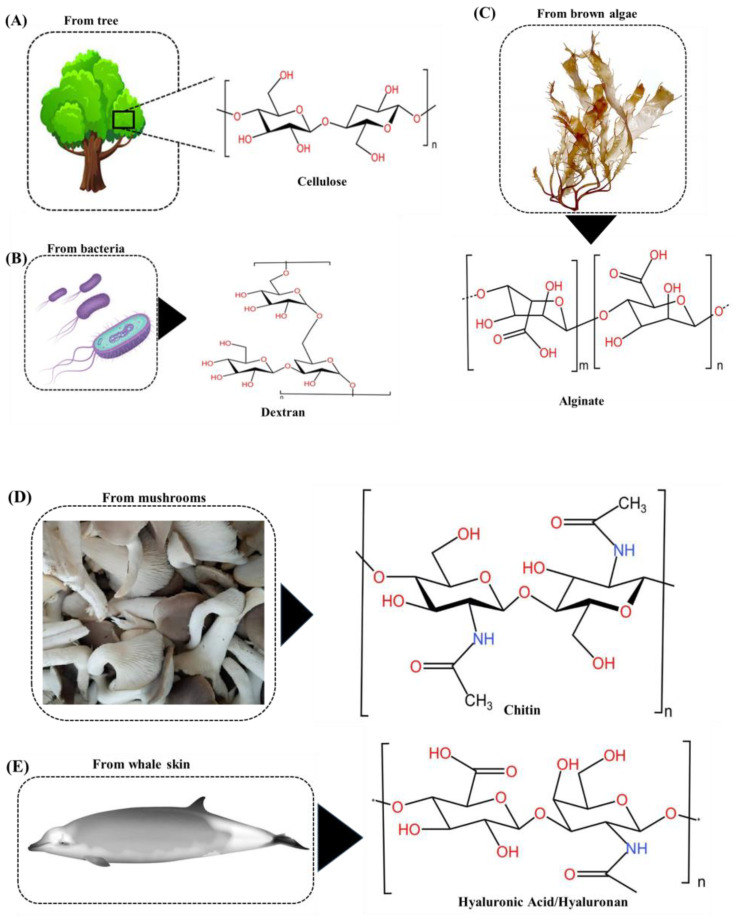
Cellulose from trees with its structure (**A**), dextran from bacteria with its structure (**B**), alginate from brown algae with its structure (**C**), chitin from mushrooms with its structure (**D**), and hyaluronic acid/hyaluronan from whale skin with its structure (**E**).

Cellulose is a polysaccharide composed of glucose units bound by β-1,4-glycosidic linkages. It is a complex carbohydrate. Cellulose is the predominant organic compound found on Earth and plays a vital role in the cell walls of plants, imparting strength and rigidity to their structure [37]. Cellulose aid as the main source of dietary fiber for both humans and animals. Cellulose is a non-soluble linear polymer that creates lengthy, straight chains that are connected to each other through hydrogen bonds, resulting in the formation of microfibrils. The microfibrils form in pairs to create macrofibrils, which provide the plant cell wall with durability and rigidity (Figure 3) [38].

The chemical composition is given by the simplified formula (β-D-glucose)n. The variable “n” in the formula denotes the quantity of glucose units that are interconnected to create the polymer [39]. The β-1,4-glycosidic bond connects the C-1 and C-4 carbon atoms of the adjoining glucose units, creating a straight chain of glucose molecules. The hydroxyl (OH) groups on each glucose unit were positioned in alternating up- and down-orientations. Cellulose is a homopolysaccharide with the chemical formula (C_6_H_10_O_5_)n. It can be hydrolyzed to produce glucose (C_6_H_12_O_6_). The number of repeated units per chain length varies depending on the source. The glucose units in cellulose are linked through hydrogen bonds in a repetitive manner. Within each repeating unit, six hydroxyl groups engage in intramolecular and intermolecular hydrogen bonding with cellulose chains, resulting in the formation of microfibrils [40,41].

However, nanocelluloses containing metal-binding groups, including sulfonate, carboxylic, and phosphate groups, can eliminate heavy metal ions from polluted water. Liu P. et al. investigated the adsorption behavior of Ag(I), Cu(II), and Fe(III) ions on four different types of nanocellulose biopolymers: sludge cellulose nanocrystals (CNC_SL_), bioethanol cellulose nanocrystals (CNC_BE_), phosphorylated cellulose nanocrystals from sludge (phos-CNC_SL_), and phos-CNF_SL_ [42]. The plant polysaccharides could exert antitumor activity through different mechanisms, which included preventing oncogenesis, improving immune response, inducing tumor cells apoptosis, and inhibiting tumor cell proliferation [43]. Cellulose, a naturally occurring polysaccharide derived from plants and trees, is recognized as a biodegradable and environmentally sustainable resource within the natural world. Cellulose molecules possess numerous active hydroxyl groups that demonstrate the potential for substitution with alternative functional groups [44,45]. The adsorption capacity of heavy metal ions has been shown to be around 40–80% higher in modified cellulose as compared to unmodified cellulose [46,47]. For example, cellulose that underwent modification with a sulfo group exhibited a higher adsorption capacity compared to cellulose that was not treated [48,49]. In general, the process of modifying and functionalizing cellulose can effectively eliminate several heavy metal ions concurrently. Wastewater contains a diverse range of heavy metal ions [50]. Several reports have been published regarding this research. For example, Guclu et al. (2003) [51] identified four distinct categories of cellulose graft copolymers that effectively removed Pb^2+^, Cu^2+^, and Cd^2+^ from aqueous solutions. The removal of Cd^2+^ and Cu^2+^ from aqueous solutions has been observed in cellulosic materials that incorporate grafted polyacrylonitrile and poly (acrylic acid) molecules [52]. Recently, researchers have reported the use of modified cellulose hydrogels for adsorbing heavy metal ions through an ion-exchange mechanism [53]. The modified cellulose hydrogels exhibited maximal absorption capacities of 157.51, 393.28, and 289.97 mg g^−1^ for Cu^2+^, Pb^2+^, and Cd^2+^, respectively. The adsorption of heavy metal ions via chemisorption has been observed in a cellulose nanofiber membrane functionalized with thiol groups [54]. Langmuir isotherm analysis revealed adsorption capabilities of 49.0 mg g^−1^ for Cu^2+^, 45.9 mg g^−1^ for Cd^2+^, and 22.0 mg g^−1^ for Pb^2+^.

#### 2.2.2. Dextran

Polysaccharides like dextran are abundant sources of chemically diverse and functionally distinct chemical components. They also have the added benefit of being easily modified through simple chemical processes. Dextran is a group of neutral polysaccharides that have a complicated structure consisting of around 5–10% branching units linked together through (1,3) α-linkages. Additionally, it has a main linear backbone made up of (1,6) α-D-glycoside residues. The compound originated from bacterial sources and contains three hydroxyl functional groups per glucose molecule (Figure 3) [55], which are conducive to the interaction with dyes and heavy metal cations. Dextran-graft-poly(hydroxyethyl methacrylate) gels were used as a biosorbent to eliminate dye and heavy metal cations [55]. The investigation focused on the removal of copper ions (Cu^2+^) through the utilization of water-soluble porphyrins and dextran. The rapid removal of Cu^2+^ can be achieved through the utilization of dextran, resulting in the formation of a [(TMPyP) Cu]^4+^ complex. The addition of acetone further enhances the removal efficiency, enabling the removal of nearly 100% Cu^2+^ [56]. AD-MPDVBs, nanoparticles coated with submicron aminodextran and magnetic poly(divinylbenzene), were synthesized as magnetic adsorbents to eliminate Cu(II), Pb(II), and Zn(II) ions from water-based solutions. The AD-MPDVBs demonstrated a notable and rapid capacity to adsorb Cu(II), Pb(II), and Zn(II) ions, with equilibrium occurring within a time frame of 30 min. Equilibrium investigations revealed that the adsorption of Cu(II), Pb(II), and Zn(II) adhered to the Langmuir isotherm model. The AD-MPDVBs exhibited maximum sorption capacities of 11.62, 100, and 40 mg/g for Cu^2+^, Pb^2+^, and Zn^2+^, respectively [57].

#### 2.2.3. Alginate

Sodium alginate is a naturally occurring polysaccharide that can be derived from brown algae. Sodium alginate possesses unique characteristics such as strong biocompatibility, biodegradability, and renewability. Furthermore, many of the hydroxyl and carboxyl groups exhibit a strong attraction towards heavy metal ions, making them highly adsorbent. Nevertheless, sodium alginate exhibits relatively low mechanical strength, stability, and heat resistance (Figure 3) [58,59,60]. Hence, physical or chemical alteration is typically employed to improve its suitability for heavy metal adsorption. Traditionally, the techniques used to modify sodium alginate-based adsorbents include surface grafting, cross-linking, and combining with other materials to form composites [61,62]. Surface grafting primarily improves the specificity for desired metal ions and raises the capacity to absorb metals, while cross-linking can modify chemical resistance and mechanical strength [63]. Blending sodium alginate with other substances can enhance both the adsorption capabilities and the physical characteristics of the composite materials [64,65]. A study demonstrated the efficacy of cobalt ferrite nanoparticles (CF), titanate nanotubes (T), and their alginate-based nanocomposites (CF/G, T/G) as highly suitable materials for adsorbing metal ions. The adsorbents produced exhibited removal efficiencies ranging from 60% to 100% for Fe^3+^, Cu^2+^, and As^3+^ ions under the specified circumstances. Both CF and T achieved the complete elimination of Fe^3+^ at a pH of 6.5 and after a treatment duration of 2 h [66]. Papageorgiou et al. [67] investigated the process of sequestering Cu^2+^ and Cd^2+^ ions through biosorption onto calcium alginate beads. They found that sorption occurs through a competitive mechanism in solutions containing several metals. Nevertheless, the elimination of heavy metals was mostly accomplished by adsorption onto alginate beads.

#### 2.2.4. Chitin

Chitin is a homopolymer polysaccharide composed of [poly-β-(1,4)-N-acetyl-ᴅ-glucosamine]. It is present in the cell walls of fungi, yeast, and other invertebrates, such as shrimps and crabs (Figure 3) [68]. Chitin is present in the exoskeletons of crabs and prawns as well as in the intricate structure of marine sponges belonging to the Verongida order [69]. Ehrlich et al. were the pioneers in extracting chitin from sea sponges using a process involving alternating incubation in a weak sodium hydroxide solution and acetic acid [70]. The chitin of pink shrimp was shown to absorb Pb^2+^ from aqueous solutions [71]. The highest level of adsorption was determined to be 99.7% at a pH of 9, after a contact period of 200 min, using a biosorption dosage of 5 g/L, and an initial lead concentration of 20 mg/L. The experiment was conducted at a temperature of 30 °C with an agitation speed of 200 rpm. Xiong investigated the utilization of chitin for the adsorption of Cd^2+^ [72]. The highest level of removal was achieved at pH 5.41. A positive correlation was observed between the temperature increase from 288 to 318 K and the corresponding increase in absorption efficiency from 87.1 to 102 mg/g. The FTIR spectra obtained before and after the adsorption of Cd^2+^ indicated the participation of acetylamino and hydroxyl groups in the metal removal process. A negative value of the Gibbs free energy was determined, indicating the viability and spontaneity of the adsorption process. The measured activation energy was determined to be 63.1 kJ/mol, which falls within the range of 40–800 kJ/mol and suggests the occurrence of chemisorption. Mohan and Syed Shafi [73] conducted a study investigated the efficacy of a chitin/polyethylene glycol binary blend for Cd^2+^ removal. The change in pH from 4 to 8 was observed to have an impact on the absorption of metals. The optimal pH value was observed at pH 5.5, but pH values over 5.5 resulted in a decrease in adsorption due to the precipitation of Cd^2+^ as Cd(OH)_2_. Equilibrium was attained after a contact time of 210 min.

#### 2.2.5. Hyaluronic Acid/Hyaluronan

Hyaluronic acid is a polymer composed of D-glucuronic acid and N-acetyl-D-glucosamine units connected with β-1,4 and β-1,3 glycosidic bonds. It typically has a large molecular weight and is primarily found in its negatively charged form in the body’s natural environment [74,75,76]. It is synthesized within the plasma membrane, serves as a crucial constituent of the extracellular matrix, and plays a significant role in cell motility and proliferation. As a result, it is extensively dispersed throughout animal tissues (Figure 3) [75]. The as-synthesized Fe_3_O_4_@SiO_2_-HA microspheres can be used as an effective adsorbent for the removal of copper ions from an aqueous solution [77]. Wang investigated the efficacy of a hydrogel composed of hyaluronic acid methacrylate (HAMA) for the adsorptive removal of lead (Pb(II)) ions from aqueous solutions [78]. A suggested adsorption mechanism was presented, which was associated with the reported analytical performance of the device. This mechanism was then supported by experimental evidence. The adsorption capability of the device by hyaluronic acid was attributed to both the molecular interactions arising from the HAMA hydrogel and the electrochemical accumulation originating from the electrode beneath.

## 3. Mechanism of Heavy Metal Toxicity

### 3.1. Common Heavy Metals of Concern

#### 3.1.1. Cadmium and Its Mechanism of Toxicity

Cadmium (Cd), despite being uncommon, is naturally present in soil and minerals, including sulfides, sulphates, carbonates, chlorides, and hydroxide salts, as well as in water. Excessive concentrations of Cd in water, air, and soil may increase as a result of industrial work, leading to significant human exposure to Cd [79]. Furthermore, the ingestion of contaminated food results in substantial Cd exposure. Exposure to Cd can also occur through smoking, which can increase the levels of Cd in the blood and urine. The occurrence of Cd in polluted water has the potential to disrupt essential body functions, which may lead to both immediate and prolonged disease [80,81,82]. It is a residual product of zinc manufacturing that can potentially expose humans and animals to occupational or natural environments. Following human absorption, this metal progressively accumulates within the body over the course of its lifetime. Originally, it was developed as a tin substitute during World War I; this metal was also applied in the paint industry as a pigment. Workers may be exposed to Cd in industries such as alloy, battery, and glass production, as well as electroplating. Given the significance of this topic, certain countries regularly conduct air monitoring to assess the concentration of Cd [83]. Plants slowly absorb this metal from the soil, accumulating and concentrating it; Cd then moves along the food chain, ultimately reaching the human body. Cd pollution has been detected in rice, wheat, and seafood [84]. Research studies have indicated that in China, the cumulative extent of land contaminated by Cd exceeds 27,181.000 acres, and the yearly quantity of Cd industrial waste released into the environment is estimated to exceed 680 tons. Environmental Cd exposure is significantly greater in Japan and China than in other countries [85]. Cadmium is a highly poisonous heavy metal that is not essential for cell function. It is known to have a negative effect on the enzymatic systems of cells, causing oxidative stress and resulting in nutritional deficiencies in plants [86]. The precise mechanism of Cd toxicity remains unclear, although its impact on cellular functions is widely known [87]. The binding of cadmium to cysteine-rich proteins, such as metallothionein, results in a 3000-fold increase in the cadmium concentration. The cysteine–metallothionein complex induces hepatotoxicity in the liver and then circulates to the kidney, where it accumulates in renal tissue, causing nephrotoxicity. Cd can form complexes with cysteine, glutamate, histidine, and aspartate ligands, which can result in iron deficiency [88]. The toxic dose of cadmium in humans is influenced by factors such as the method of exposure (e.g., inhalation, ingestion, or skin contact), the length of exposure, and individual vulnerability. Cadmium is a poisonous heavy metal that builds up in the body, mainly in the kidneys and liver, and can have harmful health consequences (Table 1). The lowest lethal dose of Cd is 5 gr in a 70 kg man [89]. Cd and Zn share identical oxidation states, allowing Cd to substitute Zn in metallothionein. Consequently, this substitution hinders the capacity of metallothionein to scavenge free radicals within the cell.

#### 3.1.2. Lead and Its Mechanism of Toxicity

Lead (Pb), a highly poisonous metal, has created significant environmental pollution and health issues worldwide owing to its ubiquitous usage. Pb is a shiny, silver-colored metal that appears slightly bluish in dry climates. Upon exposure to air, it undergoes oxidation, resulting in the formation of a diverse array of chemicals, which vary depending on specific conditions. The primary sources of Pb are gasoline and home paints. However, lead exposure has also been linked to other sources, such as lead bullets, plumbing pipes, pewter pitchers, storage batteries, toys, and faucets [90]. The environment has become contaminated by lead and its compounds as a consequence of human activities, such as mining, manufacturing, and burning fossil fuels. This contamination affects air, water, and soil. Lead is a harmful environmental contaminant that exhibits significant toxicity to several organs. Although lead can be absorbed through the skin, the majority of absorption occurs through the respiratory and digestive systems. Vehicle exhaust in the US discharges an annual amount of lead ranging from 100 to 200,000 tons. The presence of Pb in the general public is primarily attributed to either food or drinking water, as it can be absorbed by plants, fixed in the soil, and enter water bodies [91]. The internationally recognized threshold for concern regarding Pb poisoning is a blood level of 10 μg/dL [92,93].

The presence of Pb in live cells results in toxicity via both ionic mechanisms and oxidative stress. Several studies have demonstrated that oxidative stress in living cells arises from an imbalance between the formation of free radicals and the generation of antioxidants, which are responsible for neutralizing reactive substances or repairing the consequent harm.

Moreover, Pb has the possibility to disrupt the equilibrium of the antioxidant system and trigger inflammatory reactions in multiple organs. Pb exposure can cause changes in the body’s physiological system and is linked to multiple diseases (Table 1) [94,95,96].

Pb is a highly hazardous substance with detrimental effects on neurological, biological, and cognitive functions in the human body. The primary cause of lead toxicity is the capacity of lead metal ions to displace other divalent cations, such as Ca^2+^, Mg^2+^, and Fe^2+^, and monovalent cations, such as Na^+^. This disruption ultimately interferes with biological processes. The ionic mechanism of Pb toxicity induces substantial alterations in diverse biological processes, including cell adhesion, intra- and inter-cellular signaling, protein folding, maturation, apoptosis, ionic transportation, enzyme control, and the release of neurotransmitters. Even at extremely low concentrations, Pb can replace calcium and affect the function of protein kinase C, a crucial regulator of brain excitation and memory formation [97].

#### 3.1.3. Mercury and Its Mechanism of Toxicity

Mercury (Hg) is a naturally occurring metallic element with a shiny silver-white appearance in the form of a liquid. It is odorless and transforms into a colorless and odorless gas when exposed to heat. Mercury is highly poisonous, and tends to accumulate in living organisms. The presence of Hg has a negative impact on marine ecosystems; therefore, many studies have focused on the distribution of Hg in water. Mercury is present in the atmosphere, water, and soil, and can be found in three different states: elemental or metallic mercury (Hg^0^), inorganic mercury (Hg^+^, Hg^2+^), and organic mercury (typically methyl or ethyl mercury) [98]. Mercury is commonly used in thermometers, barometers, mercury arc lamps, fluorescent lamps, and catalysts. Furthermore, it has applications in the pulp and paper sectors, serves as a constituent in batteries, and is utilized in dental preparations such as amalgams. Elemental mercury exists in a liquid state at room temperature and can be easily transformed into vapor. The gaseous state of Hg is riskier than its liquid state. Container breakup leads to the release of Hg^0^, which can be lethal when inhaled in large quantities. Organic mercury compounds, like methyl mercury (Me-Hg) and ethyl mercury (Et-Hg), exhibit higher toxicity than their inorganic counterparts do. The hierarchy of Hg toxicity, in ascending order, is as follows: Hg^0^ < Hg^2+^, Hg^+^ < CH_3_-Hg [99].

Mercury is widely recognized as a dangerous metal, and its toxicity frequently leads to acute heavy metal poisoning. In 1997, the American Association of Poison Control Centers documented 3596 instances of mercury poisoning. Methylmercury is a neurotoxic compound that causes microtubule disintegration, mitochondrial damage, lipid oxidation, and the buildup of neurotoxic chemicals such as serotonin, aspartate, and glutamate [100]. According to the Environmental Protection Agency and the National Academy of Science, approximately 8–10% of American mothers possess mercury levels that would induce neurological issues in the children they give birth to [101]. Mercury has a significant impact on tertiary and quaternary protein structures, causing damage and the disruption of cellular functions. This occurs when mercury binds to selenohydryl and sulfhydryl groups, leading to reactions with methyl mercury, which impairs cellular structure. It exhibits liquid properties and is efficiently absorbed by the gastrointestinal tract, lungs, and the skin. It has the ability to traverse the placenta and enter the breast milk. Chronic exposure to a substance can lead to toxicity that affects the central nervous system (CNS) (Table 1). The symptoms evolve from paresthesia to ataxia, and then develop into generalized weakness, vision and hearing impairment, tremor, and muscle stiffness, ultimately resulting in coma and death [102].

#### 3.1.4. Arsenic and Its Mechanism of Toxicity

Arsenic is a significant heavy metal that raises concerns from both ecological and individual health perspectives [103]. Arsenic is the 20th most prevalent element on the planet, and its inorganic modifications, like arsenite and arsenate compounds, can cause damage to both the environment and living organisms (Table 1). Humans can be exposed to arsenic through natural, industrial, or unintentional sources. The contamination of drinking water can occur due to the application of arsenical pesticides, the presence of natural mineral deposits, or the disposal of arsenical substances. The intentional ingestion of arsenic in situations of suicide attempts or unintentional ingestion by children can also lead to acute poisoning [104,105]. Arsenic toxicity is a major public health concern. Humans are exposed to arsenic mostly through the ingestion of contaminated water and food as well as through occupational activities.

Characterizing arsenic as a single element is extremely challenging because of its complex chemistry and the existence of several arsenic compounds. They can exist in either trivalent or pentavalent forms and are abundant in the natural environment. Arsenic trioxide, sodium arsenite, and arsenic trichloride are the most prevalent inorganic trivalent arsenic compounds. It is widely recognized as a metalloid or pharmaceutical substance and is famously known as the monarch of toxins and toxin of monarchs [106]. Arsenic exists in the form of inorganic compounds (As^3+^ and As^5+^), organic, metalloid (As^0^), and arsine (AsH_3_), and their toxicity levels, listed in descending order, are as follows: AsH_3_ > As^3+^ > As^5+^ > As^0^ > organic arsenicals [107,108,109].

**Table 1:** Toxic mechanisms of Cd, Pb, As, and Hg.

Toxic Metal	Organ Toxicity	Disrupted Macromolecule/Mechanism of Action	References
Cadmium (Cd)	- Liver damage; - Lung injuries; - Kidney dysfunction; - Degenerative bone disease; - GI disorders; - Cancer; - Disorders in the metabolism of Zn and C.	- Endoplasmic reticulum stress; - Dysregulation of Ca, Zn, and Fe homeostasis; - Apoptosis; - miRNA expression dysregulation; - Cd-MT absorption by the kidneys; - ROS generation; - Altered phosphorylation cascades; - Low serum PTH.	[110,111,112,113]
Lead (Pb)	- Lung dysfunction; - CNS injury; - GI colic; - Hematological changes (anemia); - Liver damage; - Reduced pulmonary function; - Cardiovascular dysfunction.	- Increased serum ET-1, NO, and EPO; - Increased inflammatory cytokines IL-1β, TNF-α, and IL-6 in the CNS; - Reduced GSH, SOD, CAT, and GPx levels; - Inactivation of δ-ALAD and ferrochelatase; - (inhibition of heme biosynthesis).	[114,115,116,117]
Arsenic (As)	- CNS injury; - Skin and hair changes; - Cardiovascular dysfunction; - GI discomfort; - Liver damage.	- Uncoupler of oxidative phosphorylation (inhibition of ATP formation); - Thiol binding (GSH conjugation); - Damage of capillary endothelium; - Alterations in neurotransmitters; - Homeostasis.	[118,119,120]
Mercury (Hg)	- GI ulceration; - Renal dysfunction; - CNS injuries; - Hepatotoxicity.	- ROS production; - Enzyme inhibition; - Thiol binding (GSH conjugation); - Aquaporin mRNA reduction; - Glutathione peroxidase inhibition; - Increased c-fos expression.	[110,111,112,113]

### 3.2. Effects of Heavy Metal Toxicity on Living Organisms

**Figure 4:**
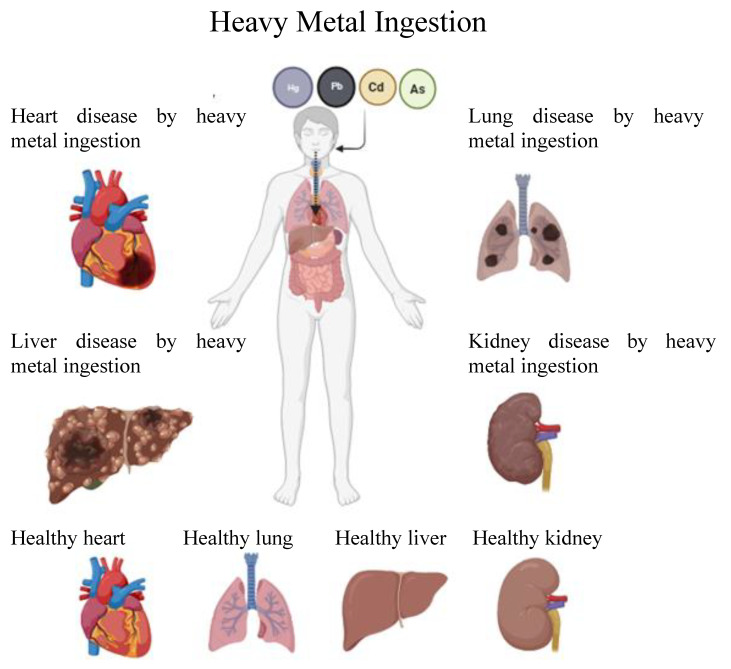
Organ toxicity following heavy metal ingestion. Created with the web-based BioRender tool (BioRender.com).

Common heavy metals, such as cadmium, lead, mercury, and arsenic, are frequently present in both the environment and food. Even at smaller doses, they are known to endanger health but are deadly in greater quantities. Heavy metal toxicity can reduce energy levels and affect the functioning of the brain, lungs, kidneys, liver, blood composition, and other critical organs (Figure 4). Continuing exposure can lead to gradual advancement of physical, muscular, and neurological degenerative processes that parallel diseases such as multiple sclerosis, Parkinson’s disease, Alzheimer’s disease, and muscular dystrophy. Frequent long-term exposure to certain metals and their compounds will cause cancer [121].

#### 3.2.1. Effects of Cadmium Toxicity on Living Organisms

Cd can result in both acute and chronic poisoning [122]. Cd exhibits a significant level of toxicity towards the kidney and tends to concentrate in the proximal tubular cells at elevated concentrations. Cd can induce bone mineralization either by bone injury or by impairing kidney function. Research conducted in both humans and animals has demonstrated that exposure to cadmium can lead to osteoporosis, which is characterized by skeletal damage. Additionally, Cd exposure can disrupt calcium metabolism, contribute to the production of renal stones, and cause hypercalciuria. Exposure to elevated amounts of Cd through inhalation can significantly harm the respiratory system. Elevated cadmium consumption can cause gastric irritation, resulting in symptoms such as vomiting and diarrhea. With prolonged exposure and lower amounts, it can accumulate in the kidneys, resulting in kidney disease, weakened bones, and lung damage [123].

#### 3.2.2. Effects of Lead Toxicity on Living Organisms

Lead toxicity, often known as lead poisoning, can manifest as either acute or chronic toxicity. Short-term exposure may result in symptoms, such as a loss of appetite, headache, high blood pressure, abdominal pain, fatigue, sleep disorders, arthritis, hallucinations, vomiting, weight reduction, difficulty passing stool, blood deficiencies, kidney failure, irritability, slowness, memory problems, and behavioral issues. A delayed development of typical childhood behaviors, like language and speech abilities, and permanent memory loss are frequently observed [124,125,126,127,128]. Children are more susceptible to lead poisoning because their small bodies undergo constant growth and development. Children exhibit a higher rate of lead absorption than adults, resulting in greater harm to the body than in older people. A prolonged exposure to lead can lead to memory loss, birth defects, psychosis, autism spectrum disorder, allergic reactions, dyslexia, weight reduction, hyperactivity, paralysis, muscle weakness, brain injury, kidney failure, and even death [129]. Acute exposure arises mainly in the workplaces and specific manufacturing industries that utilize Pb. Lead poisoning, while preventable, remains a deadly disease that can affect the common organs in the body. When the concentration of lead in the blood–brain barrier increases, the plasma membrane shifts to the interstitial spaces of the brain, leading to the appearance of edema [130]. Lead has the potential to affect all organs of the body, with a particular focus on the nervous system, followed by the bones and teeth, cardiovascular system, and reproductive system. Pb poisoning can also lead to hearing loss and tooth problems. Nevertheless, adults experience comparable health impacts to children, although with higher thresholds [94,131]. The prolonged and significant exposure of males can damage the reproductive systems in control of sperm production.

#### 3.2.3. Effects of Mercury Toxicity on Living Organisms

Mercury can amalgamate with other elements, resulting in the formation of both organic and inorganic mercury compounds. Exposure to high concentrations of metallic, organic, and inorganic mercury can harm the brain, kidneys, and growing fetus [132]. Organic mercury readily diffuses across biomembranes and, owing to its lipophilic properties, accumulates at higher levels in fatty fish species and the livers of lean fish [133]. The neurological system is highly sensitive to various forms of mercury. This substance causes disruptions to the neurological system, the impairment of brain processes, damage to DNA and chromosomes, allergic reactions leading to skin rashes, fatigue, and headaches, as well as having adverse effects on reproduction, such as sperm damage, birth deformities, and miscarriages [134]. Exposure to mercury vapors can lead to bronchitis, asthma, and respiratory problems.

#### 3.2.4. Effects of Arsenic Toxicity on Living Organisms

Arsenic is primarily absorbed by the small intestine. Exposure can also occur through direct contact with the skin or through the inhalation of the substance [135]. Arsenic is present in most paints, dyes, detergents, metals, semiconductors, and drugs. Arsenic is additionally discharged into the environment in larger quantities by specific herbicides, fertilizers, and animal feeding processes. Arsenic toxicity in over 30 nations worldwide is primarily attributed to the consumption of drinking water contaminated with arsenic [136]. Arsenite and arsenate, which are inorganic forms of arsenic, pose greater risks to human health. Arsenic can enter the human body through the inhalation of polluted air, the consumption of contaminated food, and the ingestion of polluted water. Research conducted in humans has demonstrated a clear link between drinking water contaminated with arsenic and negative outcomes during pregnancy. Arsenic has been found to freely pass through the placenta, especially in the early stages of pregnancy, resulting in spontaneous abortion, stillbirth, preterm birth, and low birth weight [137,138]. A prolonged and chronic exposure to low levels of a substance can result in the discoloration of the skin and the occurrence of small “corns or warts” on the palms, soles, and torso. The consumption of extremely elevated quantities can lead to death. Arsine is a highly effective substance that causes the devastation of red blood cells, leading to immediate symptoms such as nausea, vomiting, difficulty in breathing, and headache. Long exposure to a substance can result in the loss of life, along with the presence of hemoglobinuria, renal failure, jaundice, and anemia in circumstances where the exposure is not fatal [139].

### 3.3. Mechanism of Heavy Metal Toxicity (Oxidative Stress and DNA Damage)

#### 3.3.1. Lead-Induced Oxidative Stress and DNA Damage

The mechanism of lead-induced oxidative stress is characterized by a disruption of the equilibrium between the production and removal of reactive oxygen species (ROS) in tissues and cellular structures, resulting in harm to membranes, DNA, and proteins. The presence of double bonds in fatty acids within the cell membrane weakens the strength of the C–H bonds on the adjacent carbon atoms, facilitating the removal of hydrogen (H) atoms. Therefore, fatty acids with zero to two double bonds demonstrate higher resilience to oxidative stress than polyunsaturated fatty acids that have more than two double bonds [140]. The fundamental process responsible for lead-induced oxidative damage to membranes is linked to changes in the fatty acid makeup of the membranes [141]. The length as well as the level of unsaturation of fatty acid chains are factors that determine the susceptibility of membranes to peroxidation. The elongation of arachidonic acid produced by lead may be responsible for the increased lipid peroxidation of the membrane [142]. Thus, lead affects various membrane-related processes, including the functioning of membrane enzymes, the processes of endo- and exocytosis, the movement of solutes across the bilayer, and the transmission of signals by inducing lateral phase separation [143].

The accumulation of lead in tissues results in oxidative DNA damage such as strand breakage. However, evidence for lead-induced oxidative damage to DNA is not entirely conclusive [144]. δ Aminolevulinic acid dehydrase (ALAD) is highly susceptible to the toxic effects of Pb [145]. Exposure to Pb causes the production of reactive oxygen species (ROS) by increasing the accumulation of δ-aminolevulinic acid (ALA) [146,147] and the resultant oxidative stress [148]. The continuous treatment of rats with ALA has been found to result in an elevated level of 8-oxo-7, 8-dihydro-2-deoxyguanosine and 5-hydroxyl-2-deoxycytidine, which is understood to be responsible for the DNA damage caused by ALA [149]. Recent data indicate that Pb exposure can cause alterations in gene expression [150], and it appears to engage with zinc-binding sites on a crucial DNA-associated protein called human protamine [151].

#### 3.3.2. Cadmium-Induced Oxidative Stress and DNA Damage

Cd is a widely acknowledged environmental contaminant that has several detrimental effects on human health. It mostly affects the lungs, liver, kidneys, and testes after acute poisoning, and can cause kidney damage, immune system dysfunction, bone damage, and tumors with prolonged exposure. Reactive oxygen species (ROS) are frequently associated with the harmful health effects caused by Cd exposure. Direct proof of the formation of free radicals was presented in *Veterinary Medicine International*. Animals exposed to steep levels of Cd experience acute toxicity, and indirect evidence suggests that reactive oxygen species (ROS) have a role in chronic Cd toxicity and cancer development. Electron spin resonance spectra showed the presence of superoxide anions, hydrogen peroxide, and hydroxyl radicals formed by Cd in living organisms. This origination of reactive oxygen species is commonly followed by the activation of redox-sensitive transcription factors such as NF-κB, AP-1, and Nrf2, as well as fluctuations in the expression of genes associated with reactive oxygen species. Oxidative stress is widely acknowledged to have significant implications in cases of acute Cd poisoning. Nevertheless, it is frequently challenging to find obvious evidence of oxidative stress after prolonged and ecologically significant exposure to low levels of Cd. The changes in gene expression due to reactive oxygen species (ROS) with chronic exposure are less significant than the changes observed in acute cadmium poisoning. This is likely caused by induced adaptive mechanisms, such as the increased production of metallothionein and glutathione, as a result of prolonged exposure to Cd. In turn, these mechanisms reduce oxidative stress caused by Cd. Fluorescence probes revealed a reduced presence of reactive oxygen species (ROS) signals in cells that have been chronically altered by Cd. Cells that have developed resistance to apoptosis can multiply despite DNA damage caused by oxidative stress, which could potentially result in the development of tumors. Therefore, reactive oxygen species (ROS) are formed as a result of sudden excessive exposure to cadmium, and they are significantly involved in tissue damage. Chronic exposure to Cd leads to a decrease in the development of reactive oxygen species (ROS), but the development of Cd tolerance through abnormal gene expression plays a significant role in chronic Cd toxicity and cancer development. The fundamental mechanisms implicated in cadmium-induced carcinogenesis include the genetic control of proto-oncogenes [152], oxidative stress [153,154,155,156,157], the disruption of cadherins, the obstruction of DNA repair, and interference with apoptosis [158]. Cadmium is a toxic substance that can harm cells. It induces oxidative stress by promoting lipid oxidation and altering the amount of glutathione within cells. It affects ubiquitin/ATP-dependent proteolytic processes. Nevertheless, the precise biological pathways underlying Cd toxicity, particularly in neural cells, are poorly understood. Neuronal cells were exposed to various doses of metal ions to examine the connection between oxidative stress caused by cadmium and the ubiquitin/ATP-dependent system. This investigation showed a reduction in glutathione levels and significant increases in protein-mixed disulfides (Pr-SSGs) [159]. The testis is the primary organ affected by Cd toxicity effects. Multiple studies have demonstrated that Cd causes testicular damage in various animal species such as mice, hamsters, rabbits, guinea pigs, and dogs [160,161]. Cd has a significant impact on the weight of sex organs, which is a key signal of potential changes in androgen status [162,163]. Various causes of testicular toxicity induced by Cd have been suggested. [164] Researchers observed a rise in Cd concentration in the hypothalamus, pituitary, and testis, as well as a reduction in follicle stimulating hormone levels in the plasma of rats. These findings imply that Cd may affect the hypothalamic–pituitary–testicular axis.

#### 3.3.3. Mercury-Induced Oxidative Stress and DNA Damage

Many in vivo and in vitro investigations have indicated that the exposure of experimental animals to inorganic or organic forms of mercury leads to the development of oxidative stress. The strong attraction of mercuric ions to thiols indicates that the depletion of intracellular thiols, particularly glutathione, might directly or indirectly lead to oxidative stress in proximal tubular cells. The authors of [165] showed that administering mercury as Hg(II) to rats caused a decrease in glutathione levels and an escalation in the production of H_2_O_2_ and lipid peroxidation in the kidney mitochondria. The study also investigated the impact of Hg(II) on hydrogen peroxide production by rat kidney mitochondria, which are a primary cellular site affected by Hg(II). This impact was also studied by Lund Miller and Woods (1991) [166] within mitochondria, when provided with a respiratory chain substrate such as succinate or malate/glutamate, and an electron transport inhibitor such as antimycin A (AA) or rotenone. Hg(II) increased H_2_O_2_ formation approximately 4-fold at the uniquinone-cytochrome b region (AA-inhibited) and 2-fold at the NADH dehydrogenase region (rotenone-inhibited). These findings indicate that low concentrations of Hg(II) lead to a reduction in mitochondrial GSH and an increase in H_2_O_2_ production in kidney mitochondria when the respiratory chain electron transport is impeded. The increased production of hydrogen peroxide (H_2_O_2_) caused by mercury (Hg(II)) can result in oxidative damage to tissues, including lipid peroxidation, which is evident in cases of mercury-induced nephrotoxicity.

#### 3.3.4. Arsenic-Induced Oxidative Stress and DNA Damage

Many studies have been conducted on the heightened generation of reactive oxygen species (ROS) and the reactions of plants to metal stress [167]. In addition to changes in phosphate metabolism, oxidative stress is an additional mechanism of As poisoning in plants [168,169,170,171]. The generation of reactive oxygen species (ROS) by arsenic is widely recognized in mammalian cells [172] but there are few reports regarding the origin and process of reactive oxygen species (ROS) generation in plants. The formation of reactive oxygen species (ROS) is triggered by arsenic through the inhibition of essential enzyme systems and the occurrence of electron leakage during the conversion of As (V) to As (III). The reduction process is accompanied by the methylation of inorganic arsenic, which is driven by redox reactions. These reactions can generate reactive oxygen species (ROS) [173]. Arsenic undergoes biomethylation to produce monomethylarsonic acid (MMA), dimethylarsinic acid (DMA), tetramethyalarsonium ion (TETRA), and trimethylarsenium oxide (TMAO). Additionally, plants metabolize arsenic into arsenocholine, arsenobetaine, and arseno-sugars [174]. Dimethylarsinic acid (DMA) induces iron-dependent oxidative stress by releasing iron from ferritin. DNA damage occurs as a result of the direct generation of reactive oxygen species from DMA^3+^ [175]. In addition to methylation, the transformation of inorganic arsenic from one oxidation state to another is a significant contributor to oxidative stress in plants.

## 4. Mechanisms of Polysaccharides in Heavy Metal Protection

### 4.1. Chelation of Heavy Metals

The term “chelation” is borrowed from the Greek word “chele”, which refers to the claw of a lobster and symbolizes the idea of firmly grasping or holding onto something. The term “chelate” was first used by Sir Gilbert T. Morgan and H. D. K. Drew in 1920. The name refers to caliper-like groups that act as two associating units and attach to a central atom to form heterocyclic rings [176]. The principle of chelation is rooted in basic coordination chemistry. However, the development of an optimal chelator and chelation therapy that effectively eliminates a particular harmful metal from a specific location in the body requires a comprehensive approach to drug design. Chelating agents are substances, either organic or inorganic, that have the capacity to bind metal ions and create complex structures known as ‘chelates’. In the case of bidentate chelates, chelating agents have atoms that bind to ligands through either two covalent connections or one covalent and one coordinate linkage. The primary ligand atoms are typically sulfur (S), nitrogen (N), and oxygen (O), which are present in chemical groups such as –SH, –S-S, –NH_2_, =NH, –OH, –OPO_3_H, and >C=O. Bidentate or multidentate ligands create cyclic structures that encompass the metal ion and two ligand atoms bonded to it [177].

**Figure 5:**
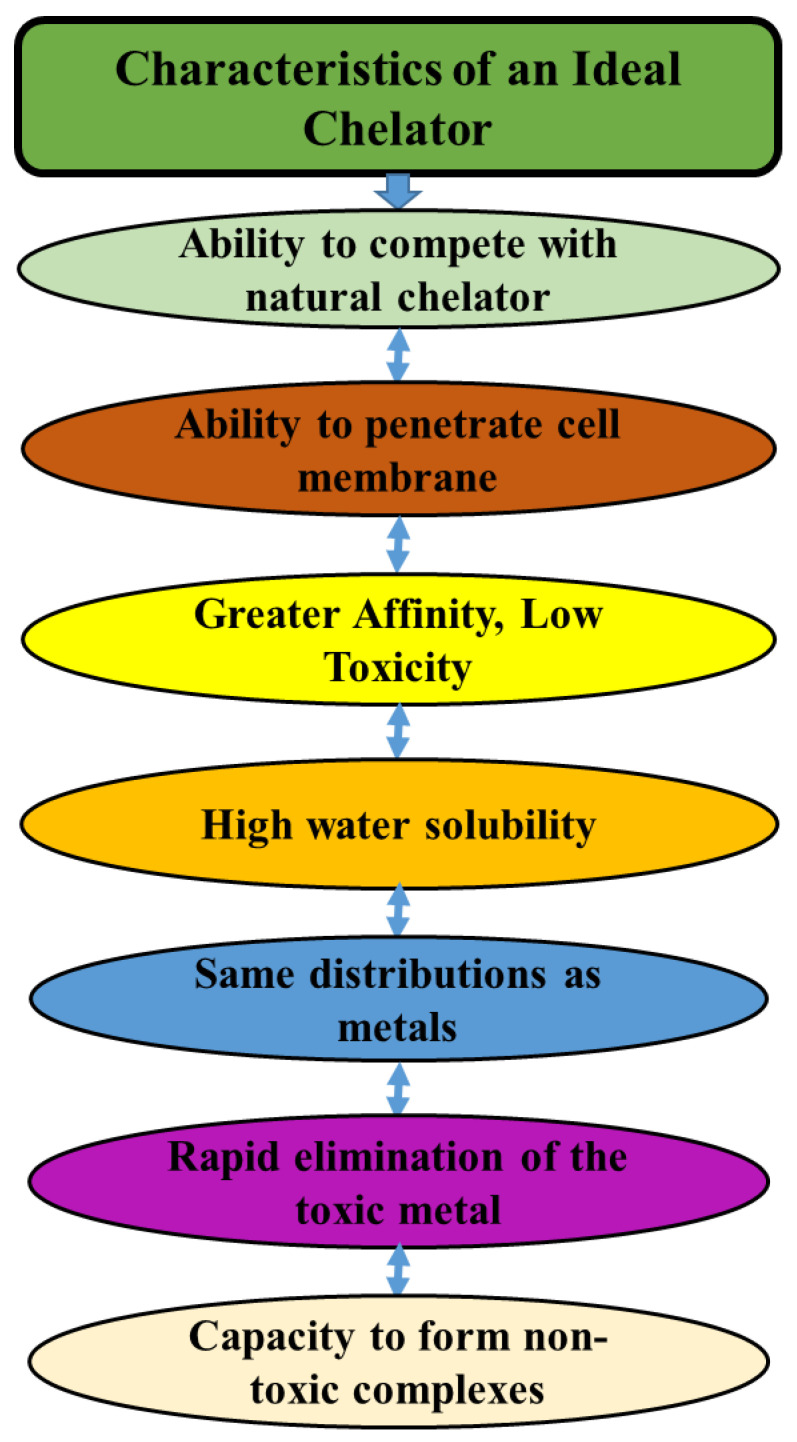
Characteristics of an ideal chelator.

An efficient chelator should possess excellent water solubility, resistance to biotransformation, and the capacity to access metal storage sites; it should also maintain chelating efficacy at the pH of bodily fluids and exhibit the ability to build metal complexes that are less hazardous than unbound metal ions (Figure 5).

Dimercaprol, also known as British anti-Lewisite or BAL, was produced as an experimental antidote to counteract the effects of Lewisite, a poisonous gas containing arsenic, during the Second World War [178]. Following World War II, a significant number of navy personnel experienced widespread lead poisoning, which was subsequently attributed to their occupation of repainted ship hulls. This establishes the medical application of EDTA as a lead chelator. BAL has been commonly used in medical prescriptions for the treatment of general metal intoxication, specifically in cases of human arsenic and mercury poisoning, because of its exceptional effectiveness. In the 1960s, BAL underwent modifications to create meso 2,3-dimercaptosuccinic acid (DMSA), a dithiol compound that has significantly reduced adverse effects. Researchers in the former Soviet Union introduced sodium 2,3-dimercaptopropane 1-sulfonate (DMPS), which is another type of dithiol, as a mercury-chelating agent. Chelation therapy has traditionally been employed to decrease the accumulation of harmful metals in the bodies of severely symptomatic patients with high biological indicators [9,10,11].

Chelating substances can influence the toxicity of metals by facilitating their movement into urine. A chelating agent, which can create a firm complex with a harmful metal, can protect biological targets from metal ions, thus decreasing their harmful effects in the surrounding area [12].

Thiolates and amines are the most suitable ligands for soft and borderline ions like Pb^2+^, Hg^2+^, Cd^2+^, and As^3+^. Therefore, it is generally observed that metal-binding sites typically consist of cysteine (Cys) or histidine (His) residues. Several of these sites have adjacent brief segments of amino acid structures that overlap between adjacent peptides with binding activity, which are employed to provisionally confirm prominent binding motifs. Most proteins and peptides involved in the uptake, transport, storage, or detoxification of necessary and unnecessary metal ions contain one or more sites where these metals can bind. The -Cys-X-X-Cys- and -Cys-Cys- motifs seen in different proteins are widely recognized for their ability to bind heavy metals [179,180]. It is widely recognized that substances containing polysaccharides can form complexes with metals by chelation. Among the functions attributed to extracellular polysaccharides, they act as natural metal chelators, thus having the ability to bind and remove metals from the human body.

### 4.2. Antioxidant Properties of Polysaccharides

Antioxidants are substances that hinder or postpone the process of oxidation in substances, even when the substance is present in a far smaller amount than the substance being oxidized [181]. Polysaccharides have emerged as crucial subjects in human diet because of their high levels of free lipid radicals. Plant materials, animal tissues, and microbes contain natural polysaccharides that shield them from oxidative stress and can be utilized for various purposes or consumed for health benefits. Polysaccharides can be extracted in pure form from source materials and utilized for applications such as food preservation, supplementation, and medicine [182]. Furthermore, plant or animal extracts that include combinations of polysaccharide compounds are also used directly to impede oxidation both in vitro and in vivo. Their high antioxidant capacity allows the removal of reactive oxygen species (ROS). Most consumers choose natural antioxidants over synthetic antioxidants for emotional reasons [183]. Polysaccharides are used in food for two main reasons: (1) to prevent the breakdown of lipids and the creation of harmful molecules called free radicals during long-term storage or when exposed to high temperatures, such as during deep-fat frying or deodorization; and (2) to decrease the levels of free radicals in the body after consuming food. The effectiveness of natural polysaccharide is determined by their specificity towards certain substrates, as well as their reliance on synergistic compounds included in both the antioxidant formulation and preserved food [184].

Organisms possess polysaccharide compounds that have demonstrated efficacy in eliminating reactive oxygen species (ROS), such as superoxide, anions, hydrogen peroxide, and hydroxyl radicals from the body. ROS are strong correlated with cardiovascular diseases, cancer, and many neurological illnesses. Polyenoic fatty acids are the primary contributors to the generation of free radicals through oxidation processes. Polysaccharides have produced significant effects in the field of human nutrition because of the presence of elevated levels of free lipid radicals, both in food and the body generated during heavy metal-induced oxidative stress following food consumption.

In recent years, numerous antioxidant polysaccharide biomolecules are being preferred as natural antioxidant medicines in foods and pharmaceuticals over the synthetic antioxidants for aesthetic grounds [185,186].

### 4.3. Stimulation of the Immune System

Polysaccharides, by virtue of their inherent characteristics, possess the capacity to serve as pharmaceutical agents capable of modulating the immune response. Furthermore, they can be utilized with a high degree of safety, as they lack harmful properties, are compatible with living organisms, and are capable of being broken down naturally. Polysaccharides have diverse biological properties, including immunomodulatory, anti-inflammatory, antioxidant, and prebiotic effects. Polysaccharides, such as lentinan, fucoidan, inulin, and glucan, have been studied to determine their ability to modulate the immune system [187]. Polysaccharides may inhibit tumor growth or stimulate immune function in the intestinal tract, which is another immunological effect [188]. Polysaccharides enhance the human immune system by stimulating various immunological pathways. Immunity, as the body’s inherent defense mechanism, plays a crucial role in combating infectious diseases and regulating inflammation. Individuals with compromised immune systems are susceptible to a range of infections and tumors owing to weakened immune surveillance caused by low immunological function. Specific monosaccharides, such as galactose, mannose, rhamnogalacturonan-I, arabinogalactan, and uronic acid, are strongly linked with immunological enhancement. Chemical alterations of polysaccharides can enhance their biological activity and potentially generate novel functionalities [28].

The incorporation of sulphate, selenium, and acetyl groups into polysaccharides alters their structures, facilitating their cellular uptake by the immune system and potentially inducing diverse immunological stimulation reactions [189,190,191]. Polysaccharides derived from the medicinal plant *Tinospora cordifolia* have distinct qualities that enhance the immune system. The α-1,4-glucan structural form activates various subsets of lymphocytes, including natural killer (NK) cells, T cells, and B cells [192].

### 4.4. Modulation of the Gut Microbiota against Heavy Metal Toxicity

The human gut microbiota, consisting of 400–1000 bacterial species, play a vital role in sustaining human health. They perform various important functions, such as food metabolism, the preservation of mucosal integrity, the modulation of the immune system, and protection against infections [193,194]. Oral exposure to heavy metals, such as lead, can cause an imbalance in the gut microbiota of both animals and humans, leading to dysbiosis. Several studies have shown corroborating evidence that an imbalance in gut microbes, known as gut microbial dysbiosis, might hinder the integrity of the intestinal mucosal barrier and the immune system of the gut mucosa. This imbalance has the potential to lead to serious gastrointestinal infections, diarrhea, and colon inflammation [195,196,197]. In addition, it can have detrimental effects that go beyond the gastrointestinal tract and lead to extraintestinal disorders such as autism, diabetes, obesity, and non-alcoholic fatty liver disease (NAFLD) [198,199,200]. Gut microbial dysbiosis is recognized as a growing contributor to the development of several diseases due to its systemic consequences [201].

Currently, polysaccharides derived from animals and plants have demonstrated significant potential as antioxidants [202,203]. Seaweed polysaccharide has gained significant interest due to its numerous health benefits for the host [204,205]. Multiple research studies have demonstrated that seaweed polysaccharide exhibits anti-inflammatory, antiviral, immunomodulatory, and anti-tumor properties [206]. Furthermore, current studies on seaweed polysaccharides have demonstrated their crucial functions in gastrointestinal sickness and its ability to enhance antioxidant capacity [207]. Polysaccharides can contribute to modulating brain impulses by the microbiota–gut–brain axis [208]. There is a growing body of evidence that demonstrates the beneficial impact of seaweed polysaccharide on the health of the host. Seaweed polysaccharide has a protective effect and can mitigate the disruption of gut microbial equilibrium induced by heavy metal toxicity.

## 5. Conclusions and Future Perspectives

Increasing levels of heavy metal pollution in soil and water cause significant concern because they have numerous harmful effects on both human health and the environment. Wastewater containing diverse heavy metal contaminants is released into rivers, entering the ecosystem cycle and causing irreversible harm to the environment. Living organisms acquire various heavy metal ions, including cadmium, lead, mercury, and arsenic, which cannot be easily removed. This study explored the potential of natural polysaccharides, which operate through mechanisms such as chelation, antioxidant defense, and immunomodulation, to bind and alleviate heavy metal ions. Polysaccharides have functional groups that enhance their adsorption efficiency. These heavy metals pose a significant risk to human health when consumed in the food chain. Polysaccharide homopolymers can serve as eco-friendly biosorbents. A significant quantity of agricultural waste, such as rice husks or jute, serves as a source of cellulose. The introduction of various functional groups into a polysaccharide by grafting, blending, or mixing with nanoparticles enhances its sorption capabilities and mechanical strength, allowing it to undergo numerous sorption and desorption cycles. The -Cys-X-X-Cys- and -Cys-Cys- motifs found in different proteins are widely recognized for their ability to bind heavy metals. Methionine and cysteine, together with N-acetylcysteine, an acetylated form of cysteine, S-adenosylmethionine, a metabolite of methionine, α-lipoic acid, and the tripeptide glutathione (GSH), aid in the process of binding and removing metals from the human body. They are secure, cost-effective, stable, water-loving, compatible with living organisms, capable of breaking down naturally, and easily modified chemically to suit the specific needs of a diverse range of uses. Chelating agents can also protect biological targets from the harmful effects of metal ions. Polysaccharides can act as pharmacological agents and regulate immune responses. The amenability of chitosan to modification and functionalization is enabled by the presence of two reactive functional groups, NH_2_ and OH. Each modification process improves the physical and mechanical properties of the chitosan adsorbents. A chelating agent that can create a firm complex with a harmful metal can defend biological targets from metal ions, thus decreasing their harmful effects in the surrounding area. BAL has been broadly used in medical prescriptions for the treatment of general metal intoxication, specifically in cases of human arsenic and mercury poisoning, owing to its exceptional effectiveness. This review discusses the effectiveness of natural polysaccharides and the mechanisms that allow them to bind to heavy metals and remove them from the body and the environment.

### Future Perspectives

The main objective will probably be to discover and separate polysaccharides from particular natural sources that are well known for their effectiveness in treating heavy metal exposure. Efforts to improve extraction technologies are anticipated to be a crucial domain of innovation, with the goals of increasing output, maintaining bioactivity, and integrating sustainable approaches. Understanding the absorption, distribution, metabolism, and excretion of polysaccharides in the human body through bioavailability and pharmacokinetic studies is essential. These studies will offer useful insights into establishing the most effective dose regimens. Combinations of polysaccharides with other natural chemicals or conventional medicines may become a popular strategy to harness synergistic interactions. The advancement to meticulously planned clinical trials will signify a crucial stage, ascertaining the safety and effectiveness of polysaccharides in human populations and expediting their transformation into therapeutic interventions for heavy metal-related diseases. In the future, there may be a shift towards personalized medicine, where treatment plans are customized according to individual variances in reaction to polysaccharides. The incorporation of nanotechnology to enhance the transportation and effectiveness of polysaccharides could provide better stability, solubility, and precise delivery. As this sector develops, regulatory factors and the implementation of standardized protocols will become increasingly crucial, guaranteeing the safety, quality, and consistency of polysaccharide-based treatments. The expected patterns in polysaccharide research indicate a potential and dynamic future for tackling the intricate problems caused by heavy metal toxicity.

## Data Availability

No new data were created or analyzed in this study. Data sharing is not applicable to this article.

## References

[1] Bradl H. (2005). Heavy Metals in the Environment.

[2] Goyer R.A., Clarkson T.W. (2001). Toxic Agents. Toxic Effects of Metals.

[3] Young R.A. (2005). Toxicity Profiles: Toxicity Summary for Cadmium, Risk Assessment Information System.

[4] Nolan K. (2003). Copper Toxicity Syndrome. J. Orthomol. Psychiatry.

[5] Duruibe J.O., Ogwuegbu M.O.C., Egwurugwu J.N. (2007). Heavy Metal Pollution and Human Biotoxic Effects. Int. J. Phys. Sci..

[6] Yang A.M., Cheng N., Pu H.Q., Liu S.M., Li J.S., Bassig B.A., Dai M., Li H.Y., Hu X.B., Wei X. (2015). Metal Exposure and Risk of Diabetes and Prediabetes among Chinese Occupational Workers. Biomed. Environ. Sci..

[7] Kim N.H., Hyun Y.Y., Lee K.B., Chang Y., Rhu S., Oh K.H., Ahn C. (2015). Environmental Heavy Metal Exposure and Chronic Kidney Disease in the General Population. J. Korean Med. Sci..

[8] Rehman K., Fatima F., Waheed I., Akash M.S.H. (2018). Prevalence of Exposure of Heavy Metals and Their Impact on Health Consequences. J. Cell. Biochem..

[9] Baum C.R. (1999). Treatment of Mercury Intoxication. Curr. Opin. Pediatr..

[10] Guldager B., Jørgensen P.J., Grandjean P. (1996). Metal Excretion and Magnesium Retention in Patients with Intermittent Claudication Treated with Intravenous Disodium EDTA. Clin. Chem..

[11] Fournier L., Thomas G., Garnier R., Buisine A., Houze P., Pradier F., Dally S. (1988). 2,3-Dimercaptosuccinic Acid Treatment of Heavy Metal Poisoning in Humans. Med. Toxicol. Advers. Drug Exp..

[12] Andersen O. (1989). Oral Cadmium Exposure in Mice: Toxicokinetics and Efficiency of Chelating Agents. Crit. Rev. Toxicol..

[13] Lewis A.E. (2010). Review of Metal Sulphide Precipitation. Hydrometallurgy.

[14] Fu F., Wang Q. (2011). Removal of Heavy Metal Ions from Wastewaters: A Review. J. Environ. Manag..

[15] Bejan D., Bunce N.J. (2015). Acid Mine Drainage: Electrochemical Approaches to Prevention and Remediation of Acidity and Toxic Metals. J. Appl. Electrochem..

[16] Malik L.A., Bashir A., Qureashi A., Pandith A.H. (2019). Detection and Removal of Heavy Metal Ions: A Review. Environ. Chem. Lett..

[17] Mobasherpour I., Salahi E., Asjodi A. (2014). Research on the Batch and Fixed-Bed Column Performance of Red Mud Adsorbents for Lead Removal. Soil Water.

[18] Zhu Y.M., Pan L.C., Zhang L.J., Yin Y., Zhu Z.Y., Sun H.Q., Liu C.Y. (2020). Chemical Structure and Antioxidant Activity of a Polysaccharide from Siraitia Grosvenorii. Int. J. Biol. Macromol..

[19] Song Q., Zhu Z. (2020). Using: Cordyceps Militaris Extracellular Polysaccharides to Prevent Pb^2+^-Induced Liver and Kidney Toxicity by Activating Nrf2 Signals and Modulating Gut Microbiota. Food Funct..

[20] Gu S.S., Sun H.Q., Zhang X.L., Huang F.N., Pan L.C., Zhu Z.Y. (2021). Structural Characterization and Inhibitions on α-Glucosidase and α-Amylase of Alkali-Extracted Water-Soluble Polysaccharide from Annona Squamosa Residue. Int. J. Biol. Macromol..

[21] Choma A., Nowak K., Komaniecka I., Waśko A., Pleszczyńska M., Siwulski M., Wiater A. (2018). Chemical Characterization of Alkali-Soluble Polysaccharides Isolated from a *Boletus edulis* (Bull.) Fruiting Body and Their Potential for Heavy Metal Biosorption. Food Chem..

[22] Liu Y., Li H., Fan C., Min W., Gao Y. (2020). Optimization of Adsorption Process and Characterization of Cu^2+^ and Cd^2+^ by Athelia Rolfsii Polysaccharide. Sci. Technol. Food Ind..

[23] Nowak K., Wiater A., Choma A., Wiącek D., Bieganowski A., Siwulski M., Waśko A. (2019). Fungal (1 → 3)-α-D-Glucans as a New Kind of Biosorbent for Heavy Metals. Int. J. Biol. Macromol..

[24] Hu X., Gu H., Zang T., Jin Y., Qu J. (2014). Biosorption Mechanism of Cu^2+^ by Innovative Immobilized Spent Substrate of Fragrant Mushroom Biomass. Ecol. Eng..

[25] Hou W., Ma Z., Sun L., Han M., Lu J., Li Z., Mohamad O.A., Wei G. (2013). Extracellular Polymeric Substances from Copper-Tolerance Sinorhizobium Meliloti Immobilize Cu^2+^. J. Hazard. Mater..

[26] Ye S., Zhang M., Yang H., Wang H., Xiao S., Liu Y., Wang J. (2014). Biosorption of Cu^2+^, Pb^2+^ and Cr^6+^ by a Novel Exopolysaccharide from Arthrobacter Ps-5. Carbohydr. Polym..

[27] Facchi D.P., Cazetta A.L., Canesin E.A., Almeida V.C., Bonafé E.G., Kipper M.J., Martins A.F. (2018). New Magnetic Chitosan/Alginate/Fe_3_O_4_@SiO_2_ Hydrogel Composites Applied for Removal of Pb(II) Ions from Aqueous Systems. Chem. Eng. J..

[28] Xu Y., Wu Y., Sun P., Zhang F., Linhardt R.J., Zhang A. (2019). Chemically Modified Polysaccharides: Synthesis, Characterization, Structure Activity Relationships of Action. Int. J. Biol. Macromol..

[29] Cui S.W. (2005). Food Carbohydrates: Chemistry, Physical Properties, and Applications.

[30] Diener M., Adamcik J., Sánchez-Ferrer A., Jaedig F., Schefer L., Mezzenga R. (2019). Primary, Secondary, Tertiary and Quaternary Structure Levels in Linear Polysaccharides: From Random Coil, to Single Helix to Supramolecular Assembly. Biomacromolecules.

[31] Ngwuluka N.C. (2018). Responsive Polysaccharides and Polysaccharides-Based Nanoparticles for Drug Delivery. Stimuli Responsive Polymeric Nanocarriers for Drug Delivery Applications: Volume 1: Types and Triggers.

[32] Torres F.G., Troncoso O.P., Pisani A., Gatto F., Bardi G. (2019). Natural Polysaccharide Nanomaterials: An Overview of Their Immunological Properties. Int. J. Mol. Sci..

[33] Liu Z., Jiao Y., Wang Y., Zhou C., Zhang Z. (2008). Polysaccharides-Based Nanoparticles as Drug Delivery Systems. Adv. Drug Deliv. Rev..

[34] Rajalekshmy G.P., Lekshmi Devi L., Joseph J., Rekha M.R. (2019). An Overview on the Potential Biomedical Applications of Polysaccharides. Functional Polysaccharides for Biomedical Applications.

[35] Guo H., Zhang W., Jiang Y., Wang H., Chen G., Guo M. (2019). Physicochemical, Structural, and Biological Properties of Polysaccharides from Dandelion. Molecules.

[36] Michaud P. (2018). Polysaccharides from Microalgae, What’s Future?. Adv. Biotechnol. Microbiol..

[37] Sharma A., Thakur M., Bhattacharya M., Mandal T., Goswami S. (2019). Commercial Application of Cellulose Nano-Composites—A Review. Biotechnol. Rep..

[38] Liu K., Du H., Zheng T., Liu H., Zhang M., Zhang R., Li H., Xie H., Zhang X., Ma M. (2021). Recent Advances in Cellulose and Its Derivatives for Oilfield Applications. Carbohydr. Polym..

[39] Kumar Gupta P., Sai Raghunath S., Venkatesh Prasanna D., Venkat P., Shree V., Chithananthan C., Choudhary S., Surender K., Geetha K. (2019). An Update on Overview of Cellulose, Its Structure and Applications. Cellulose.

[40] Waliszewska B., Mleczek M., Zborowska M., Goliński P., Rutkowski P., Szentner K. (2019). Changes in the Chemical Composition and the Structure of Cellulose and Lignin in Elm Wood Exposed to Various Forms of Arsenic. Cellulose.

[41] Oehme D.P., Yang H., Kubicki J.D. (2018). An Evaluation of the Structures of Cellulose Generated by the CHARMM Force Field: Comparisons to in Planta Cellulose. Cellulose.

[42] Liu P., Borrell P.F., Božič M., Kokol V., Oksman K., Mathew A.P. (2015). Nanocelluloses and Their Phosphorylated Derivatives for Selective Adsorption of Ag^+^, Cu^2+^ and Fe^3+^ from Industrial Effluents. J. Hazard. Mater..

[43] Hou C., Yin M., Lan P., Wang H., Nie H., Ji X. (2021). Recent Progress in the Research of *Angelica sinensis* (Oliv.) Diels Polysaccharides: Extraction, Purification, Structure and Bioactivities. Chem. Biol. Technol. Agric..

[44] Khan G.M.A., Abdullah-Al-Mamun M., Haque M.A., Rahman M.S., Shaikh H., Anis A., Al-Zahrani S.M., Alam M.S. (2017). Mechanical, Thermal and Morphological Studies of Microfibrillated Jute/PLA Biocomposites. Indian J. Fibre Text. Res..

[45] Khan G.M.A., Shaikh H., Alam M.S., Gafur M.A., Al-Zahrani S.M. (2015). Effect of Chemical Treatments on the Physical Properties of Non-Woven Jute/PLA Biocomposites. BioResources.

[46] Wu Y.L., Xu S., Wang T., Wang C.F. (2018). Enhanced Metal Ion Rejection by a Low-Pressure Microfiltration System Using Cellulose Filter Papers Modified with Citric Acid. ACS Appl. Mater. Interfaces.

[47] Yari M., Mohsen M.A., Khojasteh F., Moradi O. (2007). Heavy Metals (Cr (VI), Cd (II) and Pb (II)) Ions Removal by Modified Jute: Characterization and Modeling. J. Phys. Theor. Chem..

[48] Donia A.M., Yousif A.M., Atia A.A., Abd El-Latif H.M. (2014). Preparation and Characterization of Modified Cellulose Adsorbents with High Surface Area and High Adsorption Affinity for Hg(II). J. Dispers. Sci. Technol..

[49] Hokkanen S., Bhatnagar A., Sillanpää M. (2016). A Review on Modification Methods to Cellulose-Based Adsorbents to Improve Adsorption Capacity. Water Res..

[50] Pohl A. (2020). Removal of Heavy Metal Ions from Water and Wastewaters by Sulfur-Containing Precipitation Agents. Water Air Soil Pollut..

[51] Güçlü G., Gürdaǧ G., Özgümüş S. (2003). Competitive Removal of Heavy Metal Ions by Cellulose Graft Copolymers. J. Appl. Polym. Sci..

[52] Okieimen F.E., Sogbaike C.E., Ebhoaye J.E. (2005). Removal of Cadmium and Copper Ions from Aqueous Solution with Cellulose Graft Copolymers. Sep. Purif. Technol..

[53] Zhao B., Jiang H., Lin Z., Xu S., Xie J., Zhang A. (2019). Preparation of Acrylamide/Acrylic Acid Cellulose Hydrogels for the Adsorption of Heavy Metal Ions. Carbohydr. Polym..

[54] Choi H.Y., Bae J.H., Hasegawa Y., An S., Kim I.S., Lee H., Kim M. (2020). Thiol-Functionalized Cellulose Nanofiber Membranes for the Effective Adsorption of Heavy Metal Ions in Water. Carbohydr. Polym..

[55] Amini-Fazl M.S., Ahmari A. (2019). Dextran-Graft-Poly(Hydroxyethyl Methacrylate) Biosorbents for Removal of Dyes and Metal Cations. Mater. Res. Express.

[56] Li K., Satoh M., Tagaya M., Kobayashi T. (2015). Coprecipitated Removal of Cu^2+^ Using Dextran in Cationic Porphyrin Aqueous Solution. Sens. Mater..

[57] Chaabouni A., Marzougui Z., Elleuch B., Eissa M.M., Elaissari A. (2013). Aminodextran Magnetic Colloidal Particles for Heavy Metals Removal. Sci. Adv. Mater..

[58] Gao X., Zhang Y., Zhao Y. (2017). Biosorption and Reduction of Au (III) to Gold Nanoparticles by Thiourea Modified Alginate. Carbohydr. Polym..

[59] Wang S., Vincent T., Faur C., Guibal E. (2018). A Comparison of Palladium Sorption Using Polyethylenimine Impregnated Alginate-Based and Carrageenan-Based Algal Beads. Appl. Sci..

[60] Ates B., Koytepe S., Ulu A., Gurses C., Thakur V.K. (2020). Chemistry, Structures, and Advanced Applications of Nanocomposites from Biorenewable Resources. Chem. Rev..

[61] Thakur S., Sharma B., Verma A., Chaudhary J., Tamulevicius S., Thakur V.K. (2018). Recent Progress in Sodium Alginate Based Sustainable Hydrogels for Environmental Applications. J. Clean. Prod..

[62] He J., Chen J.P. (2014). A Comprehensive Review on Biosorption of Heavy Metals by Algal Biomass: Materials, Performances, Chemistry, and Modeling Simulation Tools. Bioresour. Technol..

[63] Jobby R., Jha P., Yadav A.K., Desai N. (2018). Biosorption and Biotransformation of Hexavalent Chromium [Cr(VI)]: A Comprehensive Review. Chemosphere.

[64] Pan L., Wang Z., Zhao X., He H. (2019). Efficient Removal of Lead and Copper Ions from Water by Enhanced Strength-Toughness Alginate Composite Fibers. Int. J. Biol. Macromol..

[65] Wen R., Tu B., Guo X., Hao X., Wu X., Tao H. (2020). An Ion Release Controlled Cr(VI) Treatment Agent: Nano Zero-Valent Iron/Carbon/Alginate Composite Gel. Int. J. Biol. Macromol..

[66] Esmat M., Farghali A.A., Khedr M.H., El-Sherbiny I.M. (2017). Alginate-Based Nanocomposites for Efficient Removal of Heavy Metal Ions. Int. J. Biol. Macromol..

[67] Papageorgiou S.K., Katsaros F.K., Kouvelos E.P., Kanellopoulos N.K. (2009). Prediction of Binary Adsorption Isotherms of Cu^2+^, Cd^2+^ and Pb^2+^ on Calcium Alginate Beads from Single Adsorption Data. J. Hazard. Mater..

[68] Schleuter D., Günther A., Paasch S., Ehrlich H., Kljajić Z., Hanke T., Bernhard G., Brunner E. (2013). Chitin-Based Renewable Materials from Marine Sponges for Uranium Adsorption. Carbohydr. Polym..

[69] Kurita K. (2001). Controlled Functionalization of the Polysaccharide Chitin. Prog. Polym. Sci..

[70] Ehrlich H., Maldonado M., Spindler K.D., Eckert C., Hanke T., Born R., Goebel C., Simon P., Heinemann S., Worch H. (2007). First Evidence of Chitin as a Component of the Skeletal Fibers of Marine Sponges. Part I. Verongidae (Demospongia: Porifera). J. Exp. Zool. Part B Mol. Dev. Evol..

[71] Forutan R., Ehsandoost E., Hadipour S., Mobaraki Z., Saleki M., Mohebbi G. (2016). Kinetic and Equilibrium Studies on the Adsorption of Lead by the Chitin of Pink Shrimp (*Solenocera melantho*). Entomol. Appl. Sci. Lett..

[72] Xiong C. (2010). Adsorption of Cadmium (II) by Chitin. J. Chem. Soc. Pak..

[73] Rodríguez M., Zalba M., Goitía M., Pugliese A., Debbaudt A., Agulló E., Schulz P., Albertengo L. (2012). Calcareous chitin: A novel low-cost sorbent for cadmium (II). Latin Am. Appl. Res..

[74] Luo Y., Kirker K.R., Prestwich G.D. (2000). Cross-Linked Hyaluronic Acid Hydrogel Films: New Biomaterials for Drug Delivery. J. Control. Release.

[75] Tripodo G., Trapani A., Torre M.L., Giammona G., Trapani G., Mandracchia D. (2015). Hyaluronic Acid and Its Derivatives in Drug Delivery and Imaging: Recent Advances and Challenges. Eur. J. Pharm. Biopharm..

[76] Varghese O.P., Kisiel M., Martinez-Sanz E., Ossipov D.A., Hilborn J. (2010). Synthesis of Guanidinium-Modified Hyaluronic Acid Hydrogel. Macromol. Rapid Commun..

[77] Lan S., Wu X., Li L., Li M., Guo F., Gan S. (2013). Synthesis and Characterization of Hyaluronic Acid-Supported Magnetic Microspheres for Copper Ions Removal. Colloids Surfaces A Physicochem. Eng. Asp..

[78] Wang N., Bora M., Hao S., Tao K., Wu J., Hu L., Liao J., Lin S., Triantafyllou M.S., Li X. (2022). Hyaluronic Acid Methacrylate Hydrogel-Modified Electrochemical Device for Adsorptive Removal of Lead(II). Biosensors.

[79] Sheoran A.S., Sheoran V. (2006). Heavy Metal Removal Mechanism of Acid Mine Drainage in Wetlands: A Critical Review. Miner. Eng..

[80] Jiang J.H., Ge G., Gao K., Pang Y., Chai R.C., Jia X.H., Kong J.G., Yu A.C.H. (2015). Calcium Signaling Involvement in Cadmium-Induced Astrocyte Cytotoxicity and Cell Death through Activation of MAPK and PI3K/Akt Signaling Pathways. Neurochem. Res..

[81] Richter P., Faroon O., Pappas R.S. (2017). Cadmium and Cadmium/Zinc Ratios and Tobacco-related Morbidities. Int. J. Environ. Res. Public Health.

[82] Cao Z.R., Cui S.M., Lu X.X., Chen X.M., Yang X., Cui J.P., Zhang G.H. (2018). Effects of Occupational Cadmium Exposure on Workers’ Cardiovascular System. Zhonghua Lao Dong Wei Sheng Zhi Ye Bing Za Zhi.

[83] IARC (1993). Beryllium, Cadmium, Mercury, and Exposures in the Glass Manufacturing Industry.

[84] Chunhabundit R. (2016). Cadmium Exposure and Potential Health Risk from Foods in Contaminated Area, Thailand. Toxicol. Res..

[85] Han J.-X., Shang Q., Du Y. (2009). Review: Effect of Environmental Cadmium Pollution on Human Health. Health.

[86] Irfan M., Hayat S., Ahmad A., Alyemeni M.N. (2013). Soil Cadmium Enrichment: Allocation and Plant Physiological Manifestations. Saudi J. Biol. Sci..

[87] Patrick L. (2003). Toxic Metals and Antioxidants: Part II. The Role of Antioxidants in Arsenic and Cadmium Toxicity. Altern. Med. Rev..

[88] Castagnetto J.M., Hennessy S.W., Roberts V.A., Getzoff E.D., Tainer J.A., Pique M.E. (2002). MDB: The Metalloprotein Database and Browser at The Scripps Research Institute. Nucleic Acids Res..

[89] Rahimzadeh M.R., Kazemi S., Moghadamnia A.A. (2017). Cadmium Toxicity and Treatment: An Update. Casp. J. Intern. Med..

[90] Thürmer K., Williams E., Reutt-Robey J. (2002). Autocatalytic Oxidation of Lead Crystallite Surfaces. Science.

[91] Goyer R.A. (1990). Lead Toxicity: From Overt to Subclinical to Subtle Health Effects. Environ. Health Perspect..

[92] Burki T.K. (2012). Nigeria’s Lead Poisoning Crisis Could Leave a Long Legacy. Lancet.

[93] Kianoush S., Balali-Mood M., Mousavi S.R., Shakeri M.T., Dadpour B., Moradi V., Sadeghi M. (2013). Clinical, Toxicological, Biochemical, and Hematologic Parameters in Lead Exposed Workers of a Car Battery Industry. Iran. J. Med. Sci..

[94] Lanphear B.P., Hornung R., Khoury J., Yolton K., Baghurst P., Bellinger D.C., Canfield R.L., Dietrich K.N., Bornschein R., Greene T. (2005). Low-Level Environmental Lead Exposure and Children’s Intellectual Function: An International Pooled Analysis. Environ. Health Perspect..

[95] Jacobs D.E., Wilson J., Dixon S.L., Smith J., Evens A. (2009). The Relationship of Housing and Population Health: A 30-Year Retrospective Analysis. Environ. Health Perspect..

[96] Kianoush S., Balali-Mood M., Mousavi S.R., Moradi V., Sadeghi M., Dadpour B., Rajabi O., Shakeri M.T. (2012). Comparison of Therapeutic Effects of Garlic and D-Penicillamine in Patients with Chronic Occupational Lead Poisoning. Basic Clin. Pharmacol. Toxicol..

[97] Flora S.J.S., Mittal M., Mehta A. (2008). Heavy Metal Induced Oxidative Stress & Its Possible Reversal by Chelation Therapy. Indian J. Med. Res..

[98] Li R., Wu H., DIng J., Fu W., Gan L., Li Y. (2017). Mercury Pollution in Vegetables, Grains and Soils from Areas Surrounding Coal-Fired Power Plants. Sci. Rep..

[99] Kungolos A., Aoyama I., Muramoto S. (1999). Toxicity of Organic and Inorganic Mercury to Saccharomyces Cerevisiae. Ecotoxicol. Environ. Saf..

[100] Lyn P. (2002). Mercury Toxicity and Antioxidants: Part 1: Role of Glutathione and Alpha-Lipoic Acid in the Treatment of Mercury Toxicity. Altern. Med. Rev..

[101] Haley B.E. (2005). Mercury Toxicity: Genetic Susceptibility and Synergistic Effects. Med. Verit. J. Med. Truth.

[102] Clarkson T.W., Magos L. (2006). The Toxicology of Mercury and Its Chemical Compounds. Crit. Rev. Toxicol..

[103] Hughes J.P., Polissar L., Van Belle G. (1988). Evaluation and Synthesis of Health Effects Studies of Communities Surrounding Arsenic Producing Industries. Int. J. Epidemiol..

[104] Guha Mazumder D.N. (2008). Chronic Arsenic Toxicity & Human Health. Indian J. Med. Res..

[105] Saha J.C., Dikshit A.K., Bandyopadhyay M., Saha K.C. (1999). A Review of Arsenic Poisoning and Its Effects on Human Health. Crit. Rev. Environ. Sci. Technol..

[106] Gupta D.K., Tiwari S., Razafindrabe B.H.N., Chatterjee S. (2017). Arsenic Contamination from Historical Aspects to the Present. Arsenic Contamination in the Environment: The Issues and Solutions.

[107] Shah A.Q., Kazi T.G., Baig J.A., Arain M.B., Afridi H.I., Kandhro G.A., Wadhwa S.K., Kolachi N.F. (2010). Determination of Inorganic Arsenic Species (As^3+^ and As^5+^) in Muscle Tissues of Fish Species by Electrothermal Atomic Absorption Spectrometry (ETAAS). Food Chem..

[108] Sattar A., Xie S., Hafeez M.A., Wang X., Hussain H.I., Iqbal Z., Pan Y., Iqbal M., Shabbir M.A., Yuan Z. (2016). Metabolism and Toxicity of Arsenicals in Mammals. Environ. Toxicol. Pharmacol..

[109] Kuivenhoven M., Mason K. (2019). Arsenic (Arsine) Toxicity.

[110] Cheng J.P., Wang W.H., Jia J.P., Zheng M., Shi W., Lin X.Y. (2006). Expression of C-Fos in Rat Brain as a Prelude Marker of Central Nervous System Injury in Response to Methylmercury-Stimulation. Biomed. Environ. Sci..

[111] Bottino C., Vázquez M., Devesa V., Laforenza U. (2016). Impaired Aquaporins Expression in the Gastrointestinal Tract of Rat after Mercury Exposure. J. Appl. Toxicol..

[112] Chen R., Xu Y., Xu C., Shu Y., Ma S., Lu C., Mo X. (2019). Associations between Mercury Exposure and the Risk of Nonalcoholic Fatty Liver Disease (NAFLD) in US Adolescents. Environ. Sci. Pollut. Res..

[113] Zhang C., Gan C., Ding L., Xiong M., Zhang A., Li P. (2020). Maternal Inorganic Mercury Exposure and Renal Effects in the Wanshan Mercury Mining Area, Southwest China. Ecotoxicol. Environ. Saf..

[114] Struzyńska L., Da̧browska-Bouta B., Koza K., Sulkowski G. (2007). Inflammation-like Glial Response in Lead-Exposed Immature Rat Brain. Toxicol. Sci..

[115] Dongre N.N., Suryakar A.N., Patil A.J., Ambekar J.G., Rathi D.B. (2011). Biochemical Effects of Lead Exposure on Systolic & Diastolic Blood Pressure, Heme Biosynthesis and Hematological Parameters in Automobile Workers of North Karnataka (India). Indian J. Clin. Biochem..

[116] Wang J., Zhu H., Yang Z., Liu Z. (2013). Antioxidative Effects of Hesperetin against Lead Acetate-Induced Oxidative Stress in Rats. Indian J. Pharmacol..

[117] Boskabady M.H., Tabatabai S.A., Farkhondeh T. (2016). Inhaled Lead Affects Lung Pathology and Inflammation in Sensitized and Control Guinea Pigs. Environ. Toxicol..

[118] Jolliffe D.M., Budd A.J., Gwilt D.J. (1991). Massive Acute Arsenic Poisoning. Anaesthesia.

[119] Luo J.H., Qiu Z.Q., Zhang L., Shu W.Q. (2012). Arsenite Exposure Altered the Expression of NMDA Receptor and Postsynaptic Signaling Proteins in Rat Hippocampus. Toxicol. Lett..

[120] Shen S., Li X.F., Cullen W.R., Weinfeld M., Le X.C. (2013). Arsenic Binding to Proteins. Chem. Rev..

[121] Järup L. (2003). Hazards of Heavy Metal Contamination. Br. Med. Bull..

[122] Chakraborty S., Dutta A.R., Sural S., Gupta D., Sen S. (2013). Ailing Bones and Failing Kidneys: A Case of Chronic Cadmium Toxicity. Ann. Clin. Biochem..

[123] Bernard A. (2008). Cadmium & Its Adverse Effects on Human Health. Indian J. Med. Res..

[124] Cleveland L.M., Minter M.L., Cobb K.A., Scott A.A., German V.F. (2008). Lead Hazards for Pregnant Women and Children: Part 1 and Children: Part 1: Immigrants and the Poor Shoulder Most of the Burden of Lead Exposure in This Country. Part 1 of a Two-Part Article Details How Exposure Happens, Whom It Affects, and the Harm It Can Do. Am. J. Nurs..

[125] Meyer P.A., McGeehin M.A., Falk H. (2003). A Global Approach to Childhood Lead Poisoning Prevention. Int. J. Hyg. Environ. Health.

[126] Bellinger D.C. (2008). Very Low Lead Exposures and Children’s Neurodevelopment. Curr. Opin. Pediatr..

[127] Hayes A.W. (2007). Principles and Methods of Toxicology.

[128] Doull J., Casarett L.J., Klaassen C.D. (2008). Casarett and Doull’s Toxicology: The Basic Science of Poisons.

[129] Martin S.E., Griswold W. (2009). Human Health Effects of Heavy Metals: Briefs for Citizens. Environ. Sci. Technol..

[130] Teo J.G.C., Goh K.Y.C., Ahuja A., Ng H.K., Poon W.S. (1997). Intracranial Vascular Calcifications, Glioblastoma Muitiforme, and Lead Poisoning. Am. J. Neuroradiol..

[131] White L.D., Cory-Slechta D.A., Gilbert M.E., Tiffany-Castiglioni E., Zawia N.H., Virgolini M., Rossi-George A., Lasley S.M., Qian Y.C., Basha M.R. (2007). New and Evolving Concepts in the Neurotoxicology of Lead. Toxicol. Appl. Pharm..

[132] Alina M., Azrina A., Mohd Yunus A.S., Mohd Zakiuddin S., Mohd Izuan Effendi H., Muhammad Rizal R. (2012). Heavy Metals (Mercury, Arsenic, Cadmium, Plumbum) in Selected Marine Fish and Shellfish along the Straits of Malacca. Int. Food Res. J..

[133] Reilly C. (2006). Pollutants in Food -Metals and Metalloids. Mineral Components in Foods.

[134] Horowitz Y., Greenberg D., Ling G., Lifshitz M. (2002). Acrodynia: A Case Report of Two Siblings. Arch. Dis. Child..

[135] Balali-Mood M., Naseri K., Tahergorabi Z., Khazdair M.R., Sadeghi M. (2021). Toxic Mechanisms of Five Heavy Metals: Mercury, Lead, Chromium, Cadmium, and Arsenic. Front. Pharmacol..

[136] Chowdhury U.K., Biswas B.K., Chowdhury T.R., Samanta G., Mandal B.K., Basu G.C., Chanda C.R., Lodh D., Saha K.C., Mukherjee S.K. (2000). Groundwater Arsenic Contamination in Bangladesh and West Bengal, India. Environ. Health Perspect..

[137] Hopenhayn C., Ferreccio C., Browning S.R., Huang B., Peralta C., Gibb H., Hertz-Picciotto I. (2003). Arsenic Exposure from Drinking Water and Birth Weight. Epidemiology.

[138] Rahman A., Persson L.Å., Nermell B., El Arifeen S., Ekström E.C., Smith A.H., Vahter M. (2010). Arsenic Exposure and Risk of Spontaneous Abortion, Stillbirth, and Infant Mortality. Epidemiology.

[139] Fowler B.A., Moorman B., Atkins W.E., Blair P.C., Thompson M.B. (1989). Toxicity Data from Acute and Short-Term Inhalation Exposures, in American Conference of Governmental Industrial Hygenists.

[140] Halliwell B. (1989). Protection against Oxidants in Biological Systems. The Superoxide Theory of Oxygen Toxicity. Free Radic. Biol. Med..

[141] Knowles S.O., Donaldson W.E. (1990). Dietary Modification of Lead Toxicity: Effects on Fatty Acid and Eicosanoid Metabolism in Chicks. Comp. Biochem. Physiol. Part C Comp..

[142] Lawton L.J., Donaldson W.E. (1991). Lead-Induced Tissue Fatty Acid Alterations and Lipid Peroxidation. Biol. Trace Elem. Res..

[143] Adonaylo V.N., Oteiza P.I. (1999). Pb^2+^ Promotes Lipid Oxidation and Alterations in Membrane Physical Properties. Toxicology.

[144] Ye X.B., Fu H., Zhu J.L., Ni W.M., Lu Y.W., Kuang X.Y., Yang S.L., Shu B.X. (1999). A Study on Oxidative Stress in Lead-Exposed Workers. J. Toxicol. Environ. Health Part A.

[145] Farant J.P., Wigfield D.C. (1982). Biomonitoring Lead Exposure with δ-Aminolevulinate Dehydratase (ALA-D) Activity Ratios. Int. Arch. Occup. Environ. Health.

[146] Hermes-Lima M., Valle V.G.R., Vercesi A.E., Bechara E.J.H. (1991). Damage to Rat Liver Mitochondria Promoted by δ-Aminolevulinic Acid-Generated Reactive Oxygen Species: Connections with Acute Intermittent Porphyria and Lead-Poisoning. Biochim. Biophys. Acta (BBA) Bioenerg..

[147] Hermes-Lima M. (1995). How Do Ca^2+^ and 5-Aminolevulinic Acid-Derived Oxyradicals Promote Injury to Isolated Mitochondria?. Free Radic. Biol. Med..

[148] Bechara E.J.H. (1996). Oxidative Stress in Acute Intermittent Porphyria and Lead Poisoning May Be Triggered by 5-Aminolevulinic Acid. Braz. J. Med. Biol. Res..

[149] Douki T., Onuki J., Medeiros M.H.G., Bechara E.J.H., Cadet J., Di Mascio P. (1998). Hydroxyl Radicals Are Involved in the Oxidation of Isolated and Cellular DNA Bases by 5-Aminolevulinic Acid. FEBS Lett..

[150] Rossman T.G. (2000). Cloning Genes Whose Levels of Expression Are Altered by Metals: Implications for Human Health Research. Am. J. Ind. Med..

[151] Quintanilla-Vega B., Hoover D., Bal W., Silbergeld E.K., Waalkes M.P., Anderson L.D. (2000). Lead Effects on Protamine-DNA Binding. Am. J. Ind. Med..

[152] Hanahan D., Weinberg R.A. (2011). Hallmarks of Cancer: The next Generation. Cell.

[153] Thévenod F., Friedmann J.M. (1999). Cadmium-mediated Oxidative Stress in Kidney Proximal Tubule Cells Induces Degradation of Na^+^/K^+^-ATPase through Proteasomal and Endo-/Lysosomal Proteolytic Pathways. FASEB J..

[154] Piqueras A., Olmos E., Martínez-Solano J.R., Hellín E. (1999). Cd-Induced Oxidative Burst in Tobacco BY2 Cells: Time Course, Subcellular Location and Antioxidant Response. Free Radic. Res..

[155] Stohs S.J., Bagchi D., Hassoun E., Bagchi M. (2001). Oxidative Mechanisms in the Toxicity of Chromium and Cadmium Ions. J. Environ. Pathol. Toxicol. Oncol..

[156] Wätjen W., Beyersmann D. (2004). Cadmium-Induced Apoptosis in C6 Glioma Cells: Influence of Oxidative Stress. BioMetals.

[157] Ikediobi C.O., Badisa V.L., Ayuk-Takem L.T., Latinwo L.M., West J. (2004). Response of Antioxidant Enzymes and Redox Metabolites to Cadmium-Induced Oxidative Stress in CRL-1439 Normal Rat Liver Cells. Int. J. Mol. Med..

[158] Shih C.M., Ko W.C., Wu J.S., Wei Y.H., Wang L.F., Chang E.E., Lo T.Y., Cheng H.H., Chen C.T. (2004). Mediating of Caspase-Independent Apoptosis by Cadmium through the Mitochondria-ROS Pathway in MRC-5 Fibroblasts. J. Cell. Biochem..

[159] Figueiredo-Pereira M.E., Yakushin S., Cohen G. (1998). Disruption of the Intracellular Sulfhydryl Homeostasis by Cadmium- Induced Oxidative Stress Leads to Protein Thiolation and Ubiquitination in Neuronal Cells. J. Biol. Chem..

[160] Hew K.W., Ericson W.A., Welsh M.J. (1993). A Single Low Cadmium Dose Causes Failure of Spermiation in the Rat. Toxicol. Appl. Pharmacol..

[161] Xu G., Jiang X.Z. (1996). Male Reproductive Toxicity of Cadmium. Chin. J. Public Health.

[162] Biswas N.M., Gupta R.S., Chattopadhyay A., Choudhury G.R., Sarkar M. (2001). Effect of Atenolol on Cadmium-Induced Testicular Toxicity in Male Rats. Reprod. Toxicol..

[163] Laskey J.W., Phelps P.V. (1991). Effect of Cadmium and Other Metal Cations on in Vitro Leydig Cell Testosterone Production. Toxicol. Appl. Pharmacol..

[164] Lafuente A., Márquez N., Pérez-Lorenzo M., Pazo D., Esquifino A.I. (2000). Pubertal and Postpubertal Cadmium Exposure Differentially Affects the Hypothalamic-Pituitary-Testicular Axis Function in the Rat. Food Chem. Toxicol..

[165] Lund B.O., Miller D.M., Woods J.S. (1993). Studies on Hg(II)-Induced H_2_O_2_ Formation and Oxidative Stress in Vivo and in Vitro in Rat Kidney Mitochondria. Biochem. Pharmacol..

[166] Lund B.O., Miller D.M., Woods J.S. (1991). Mercury-Induced H_2_O_2_ Production and Lipid Peroxidation in Vitro in Rat Kidney Mitochondria. Biochem. Pharmacol..

[167] Gratão P.L., Polle A., Lea P.J., Azevedo R.A. (2005). Making the Life of Heavy Metal-Stressed Plants a Little Easier. Funct. Plant Biol..

[168] Hartley-Whitaker J., Ainsworth G., Meharg A.A. (2001). Copper- and Arsenate-Induced Oxidative Stress in *Holcus lanatus* L. Clones with Differential Sensitivity. Plant Cell Environ..

[169] Mascher R., Lippmann B., Holzinger S., Bergmann H. (2002). Arsenate Toxicity: Effects on Oxidative Stress Response Molecules and Enzymes in Red Clover Plants. Plant Sci..

[170] Srivastava M., Ma L.Q., Singh N., Singh S. (2005). Antioxidant Responses of Hyper-Accumulator and Sensitive Fern Species to Arsenic. J. Exp. Bot..

[171] Singh H.P., Batish D.R., Kohli R.K., Arora K. (2007). Arsenic-Induced Root Growth Inhibition in Mung Bean (*Phaseolus aureus* Roxb.) Is Due to Oxidative Stress Resulting from Enhanced Lipid Peroxidation. Plant Growth Regul..

[172] Huang C., Ke Q., Costa M., Shi X. (2004). Molecular Mechanisms of Arsenic Carcinogenesis. Mol. Cell. Biochem..

[173] Zaman K., Pardini R.S. (1996). An Overview of the Relationship between Oxidative Stress and Mercury and Arsenic. Toxic Subst. Mech..

[174] Koch I., Wang L., Ollson C.A., Cullen W.R., Reimer K.J. (2000). The Predominance of Inorganic Arsenic Species in Plants from Yellowknife, Northwest Territories, Canada. Environ. Sci. Technol..

[175] Shi H., Shi X., Liu K.J. (2004). Oxidative Mechanism of Arsenic Toxicity and Carcinogenesis. Mol. Cell. Biochem..

[176] Morgan G.T., Drew H.D.K. (1920). Researches on Residual Affinity and Co-Ordination. Part II. Acetylacetones of Selenium and Tellurium. J. Chem. Soc. Trans..

[177] Andersen O. (1999). Principles and Recent Developments in Chelation Treatment of Metal Intoxication. Chem. Rev..

[178] Aronson J.K. (2016). Meyler’s Side Effects of Drugs (Sixteenth Edition): The International Encyclopedia of Adverse Drug Reactions and Interactions.

[179] Binz P.-A., Kägi J.H.R. (1999). Metallothionein: Molecular Evolution and Classification. Metallothionein IV.

[180] Cobbett C., Goldsbrough P. (2002). Phytochelatins and Metallothioneins: Roles in Heavy Metal Detoxification and Homeostasis. Annu. Rev. Plant Biol..

[181] Halliwell B., Gutteridge J.M. (2007). Free Radicals in Biology and Medicine.

[182] Benalaya I., Alves G., Lopes J., Silva L.R. (2024). A Review of Natural Polysaccharides: Sources, Characteristics, Properties, Food, and Pharmaceutical Applications. Int. J. Mol. Sci..

[183] Pokorný J. (2007). Are Natural Antioxidants Better-and Safer-Than Synthetic Antioxidants?. Eur. J. Lipid Sci. Technol..

[184] Shabihi F. (1997). Natural Antioxidants: Chemistry, Health Effects, and Applications.

[185] Zhan K., Ji X., Luo L. (2023). Recent Progress in Research on Momordica Charantia Polysaccharides: Extraction, Purification, Structural Characteristics and Bioactivities. Chem. Biol. Technol. Agric..

[186] Wu H., Sang S., Weng P., Pan D., Wu Z., Yang J., Liu L., Farag M.A., Xiao J., Liu L. (2023). Structural, Rheological, and Gelling Characteristics of Starch-Based Materials in Context to 3D Food Printing Applications in Precision Nutrition. Compr. Rev. Food Sci. Food Saf..

[187] Cheong K.L., Yu B., Teng B., Veeraperumal S., Xu B., Zhong S., Tan K. (2023). Post-COVID-19 Syndrome Management: Utilizing the Potential of Dietary Polysaccharides. Biomed. Pharmacother..

[188] La Fata G., Weber P., Mohajeri M.H. (2018). Probiotics and the Gut Immune System: Indirect Regulation. Probiotics Antimicrob. Proteins.

[189] Zhang Y., Dong L., Liu L., Wu Z., Pan D., Liu L. (2022). Recent Advances of Stimuli-Responsive Polysaccharide Hydrogels in Delivery Systems: A Review. J. Agric. Food Chem..

[190] Lu W., Yang Z., Chen J., Wang D., Zhang Y. (2021). Recent Advances in Antiviral Activities and Potential Mechanisms of Sulfated Polysaccharides. Carbohydr. Polym..

[191] Ren J.L., Sun R.C., Liu C.F., Cao Z.N., Luo W. (2007). Acetylation of Wheat Straw Hemicelluloses in Ionic Liquid Using Iodine as a Catalyst. Carbohydr. Polym..

[192] Raveendran Nair P.K., Rodriguez S., Ramachandran R., Alamo A., Melnick S.J., Escalon E., Garcia P.I., Wnuk S.F., Ramachandran C. (2004). Immune Stimulating Properties of a Novel Polysaccharide from the Medicinal Plant Tinospora Cordifolia. Int. Immunopharmacol..

[193] Jandhyala S.M., Talukdar R., Subramanyam C., Vuyyuru H., Sasikala M., Reddy D.N. (2015). Role of the Normal Gut Microbiota. World J. Gastroenterol..

[194] Clemente J.C., Ursell L.K., Parfrey L.W., Knight R. (2012). The Impact of the Gut Microbiota on Human Health: An Integrative View. Cell.

[195] Liu C.S., Liang X., Wei X.H., Jin Z., Chen F.L., Tang Q.F., Tan X.M. (2019). Gegen Qinlian Decoction Treats Diarrhea in Piglets by Modulating Gut Microbiota and Short-Chain Fatty Acids. Front. Microbiol..

[196] Li A., Ding J., Shen T., Han Z., Zhang J., Abadeen Z.U., Kulyar M.F.E.A., Wang X., Li K. (2021). Environmental Hexavalent Chromium Exposure Induces Gut Microbial Dysbiosis in Chickens. Ecotoxicol. Environ. Saf..

[197] Xu Y., Jing H., Wang J., Zhang S., Chang Q., Li Z., Wu X., Zhang Z. (2022). Disordered Gut Microbiota Correlates With Altered Fecal Bile Acid Metabolism and Post-Cholecystectomy Diarrhea. Front. Microbiol..

[198] Yang G., Wei J., Liu P., Zhang Q., Tian Y., Hou G., Meng L., Xin Y., Jiang X. (2021). Role of the Gut Microbiota in Type 2 Diabetes and Related Diseases. Metabolism.

[199] Wan J., Ma J. (2022). Efficacy of Dietary Supplements Targeting Gut Microbiota in the Prevention and Treatment of Gestational Diabetes Mellitus. Front. Microbiol..

[200] Ye J., Wu Z., Zhao Y., Zhang S., Liu W., Su Y. (2022). Role of Gut Microbiota in the Pathogenesis and Treatment of Diabetes Mullites: Advanced Research-Based Review. Front. Microbiol..

[201] Crusell M.K.W., Hansen T.H., Nielsen T., Allin K.H., Rühlemann M.C., Damm P., Vestergaard H., Rørbye C., Jørgensen N.R., Christiansen O.B. (2018). Gestational Diabetes Is Associated with Change in the Gut Microbiota Composition in Third Trimester of Pregnancy and Postpartum. Microbiome.

[202] Chen F., Huang G. (2018). Preparation and Immunological Activity of Polysaccharides and Their Derivatives. Int. J. Biol. Macromol..

[203] Qiu S.-M., Veeraperumal S., Tan K., Zhong S., Cheong K.L. (2024). The in Vitro Anti-Inflammatory Mechanism of Porphyra Haitanensis Oligosaccharides on Lipopolysaccharide-Induced Injury in IEC-6 Cells. J. Funct. Foods.

[204] Lomartire S., Gonçalves A.M.M. (2022). Novel Technologies for Seaweed Polysaccharides Extraction and Their Use in Food with Therapeutically Applications—A Review. Foods.

[205] Lu S.Y., Tan K., Zhong S., Cheong K.L. (2023). Marine Algal Polysaccharides as Future Potential Constituents against Non-Alcoholic Steatohepatitis. Int. J. Biol. Macromol..

[206] Yu B., Wang M., Teng B., Veeraperumal S., Cheung P.C.K., Zhong S., Cheong K.L. (2023). Partially Acid-Hydrolyzed Porphyran Improved Dextran Sulfate Sodium-Induced Acute Colitis by Modulation of Gut Microbiota and Enhancing the Mucosal Barrier. J. Agric. Food Chem..

[207] Wang M., Veeraperumal S., Zhong S., Cheong K.L. (2023). Fucoidan-Derived Functional Oligosaccharides: Recent Developments, Preparation, and Potential Applications. Foods.

[208] Ullah S., Khalil A.A., Shaukat F., Song Y. (2019). Sources, Extraction and Biomedical Properties of Polysaccharides. Foods.

